# A Sensor Fusion Method for Pose Estimation of C-Legged Robots

**DOI:** 10.3390/s20236741

**Published:** 2020-11-25

**Authors:** Jorge De León, Raúl Cebolla, Antonio Barrientos

**Affiliations:** Centro De Automática y Robótica (UPM-CSIC), Universidad Politécnica de Madrid, Calle José Gutiérrez Abascal, 2. 28006 Madrid, Spain; raul.cebolla.arroyo@alumnos.upm.es (R.C.); antonio.barrientos@upm.es (A.B.)

**Keywords:** legged locomotion, mobile robots, robot control, robot kinematics, robot motion, robot sensing systems, robots

## Abstract

In this work the authors present a novel algorithm for estimating the odometry of “C” legged robots with compliant legs and an analysis to estimate the pose of the robot. Robots with “C” legs are an alternative to wheeled and tracked robots for overcoming obstacles that can be found in different scenarios like stairs, debris, etc. Therefore, this kind of robot has become very popular for its locomotion capabilities, but at this point these robots do not have developed algorithms to implement autonomous navigation. With that objective in mind, the authors present a novel algorithm using the encoders of the legs to improve the estimation of the robot localization together with other sensors. Odometry is necessary for using some algorithms like the Extended Kalman Filter, which is used for some autonomous navigation algorithms. Due to the flexible properties of the “C” legs and the localization of the rotational axis, obtaining the displacement at every step is not as trivial as in a wheeled robot; to solve those complexities, the algorithm presented in this work makes a linear approximation of the leg compressed instead of calculating in each iteration the mechanics of the leg using finite element analysis, so the calculus level is reduced. Furthermore, the algorithm was tested in simulations and with a real robot. The results obtained in the tests are promising and together with the algorithm and fusion sensor can be used to endow the robots with autonomous navigation.

## 1. Introduction

Legged robots have experienced a significant growth in interest during the last decade due to the natural limitation pf the ground robots with conventional systems, wheels and tracks, concerning overcoming uneven terrains or obstacles like steps.

The robots with legs are inspired in diverse types of animal species that can walk, whether biped (ATLAS [1], TEO [2]), quadruped (Cheetah [3], Anymal [4]), hexapods (LAURON [5], R-III [6]) or octopods. Nevertheless, the majority of these robots attempt replications of the morphology of the animal by which they are inspired; therefore, they try to obtain the same degrees of freedom (DoF) in each extremity, this fact leads to the robots obtaining a high complexity for the control system and its mechanics. In Table 1, a quantity of the DoF for the different legged robots is shown.

For solving both complexities, control and mechanical, in 2001 the robot called “RHex” [7] was developed. This robot is the first one that presents a configuration with six legs where each leg has a “C” shape and only one rotation DoF.

The reason to select a configuration of the hexapod robot is due to the conclusions of several biological studies of insects (cockroaches and beetles). These studies have shown that the hexapods have developed a gait pattern (*GP*) called “alternating tripod” that presents the advantage of been always statically stable [8,9,10]. On the other hand, the implementation of one leg with “C” shape and 1 DoF gives to the robot the ability to overcome obstacles but with a control and mechanical complexity much lower [11]. However, the design of the legs with a unique DoF restricts the capabilities for the displacement; therefore, this robot does not present the mobility of a holonomic robot. Even so, the results obtained for its maneuverability and displacement are excellent [12] and present complex gait patterns for overcoming stairs [13,14], pronking [15], walking with only two legs [16] or realizing backflips [17].

The development of the RHex robot was initially supported by a DARPA CBS/CBBS program and National Science Foundation grants. From this original project, different versions of the robot were designed in order to include new features, for example, to be water resistant (Rugged RHex [18], Shelly [18], AQUA [19,20]), to be used as a research platform (X-RHex [21], XRL [22], EduBOT [23], Sensor-RHex [24], MiniRHex [25]), and for work in desert environments (Desert RHex [26], SandBot [27]).

Subsequently, new robotics platforms with similar configurations were developed in other robotics laboratories and research centers: AbhisHex [28], Quattroped [29], ELHR [10], Turboquad [30,31], iRHex [32] and the robot presented in this work CLHeRo v2.5 (C-Legs HExapod RObot) [33].

However, despite the movement qualities of this robots family, some capabilities have not yet been achieved that nowadays are essentials for a mobile robot platform, like autonomous navigation. This is a largely due to the difficulty of obtaining a robust method to analyze and compute the pose and orientation of the robot. Some developments were carried out to try to estimate the displacement of the robot with proprioceptive sensors [34,35,36] or with the use of external sensors [37,38,39,40,41,42]. However, none of the previous works have made a sensor fusion with an odometry algorithm to obtain a better and more robust pose estimation. This lack of the develop of this kind of hexapod robot will be addressed in this work.

Autonomous navigation allows to guide and orientate a mobile robot to reach a desired position. To achieve this objective is necessary to endow with sensors the robot. These sensors can be implemented redundantly, complementary or both at the same time [43]. The sensor fusion is a technique that have been used for decades, and become more important when the abilities for autonomous exploration, mapping and autonomous navigation could be achieved. Some relevant articles in this field is the one by Luo and Kay [44], the report “Where Am I” [45] and the book “Integration, coordination and control of multi-sensor robot systems” [46].

**Figure 1:**
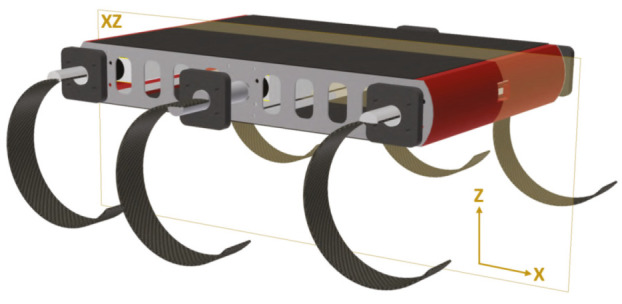
Sagittal plane of the CLHeRo.

Using the encoder sensors of the motors, like a traditional wheeled robot, to estimate the robot position using the differential drive algorithm is not possible for the “C” leg family. There are 3 main factors: the first one is that the leg is not always in contact with the ground, and therefore, the measurements are not always valid. Imagine the robot lay down on the ground and the 6 legs turning in its aerial phase, the encoder is counting, but the robot is not moving. The second point, is the movement in the XZ plane, sagittal plane (Figure 1), described by the C-legs robots is a cycloid. This kind of curve cannot be computed as the radius of the leg by the distance to the motor, it is more complex. Finally, the legs has flexible properties; therefore, the radius of the leg varies increasing or decreasing the nominal length of it.

Therefore, in this work the authors present a study of the mathematical model for obtaining the odometry of robots with “C” legs. The theoretical model developed is supported by simulations and tests with the CLHeRo robot and the ROS middleware. Moreover, the pose of the robot is compare with the measures of other external sensors and an extended Kalman filter.

In this present study, we start with the description of the platform that will be used (Section 2) and proceed with the study of the kinematic model of the robot (Section 3). Next, the solutions provided (Section 4) where the mathematical model for the odometry will be described. Finally the simulations and tests in indoor and outdoor scenarios with the real robot will be explained (Section 5) and the work conclusions (Section 6).

## 2. Robotic Platform

**Figure 2:**
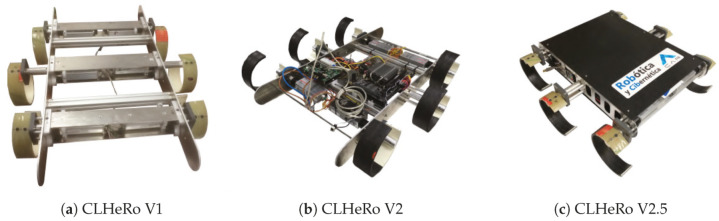
The three versions of the CLHeRo robot.

The CLHeRo V2.5 is an autonomous robot for Search and Rescue tasks inspired in the RHex robots family, designed and built by Robotics and Cybernetics Group of the Centre for Automation and Robotics (UPM-CSIC) [33,47,48,49]. The robot has two main components: the chassis and the legs. The chassis is composed by two lateral frames connected between them, inside, all the electronics components are enclosures. The legs are located in the external sides of the chassis, like any hexapod insect. The actual design of the robot can be seen in the Figure 2c, together with the prior versions (Figure 2a,b). This is the third version for the CLHeRo platform, in this new design we have introduce some improvements in order to make its construction simpler but at the same time as robust as the original version. With that in mind, we have try, whenever possible, select components that can be purchased instead of having to manufacture the pieces with specific machines or complicated and delicate processes. The robot’s body is compact and thin in profile, as strength as the original CLHeRo and very similar to the RHex platforms.

The robot legs only have 1 DoF of rotation and each one is actuated by a Maxon Compact Drive (MCD), with a nominal voltage between 12 volts and 50 volts. The MCD has coupled a planetary gearbox (33:1) that permits to achieve 3.5 N/m and a maximum of 12,000/33 RPM. Communications between the main computer and the main motor control modules operate over USB/RS232 adapter, but the motors units are connected to one another with CanOpen protocol. In contrast with prior RHex platforms and similar robots, our robot control software uses the ROS framework [50].

**Figure 3:**
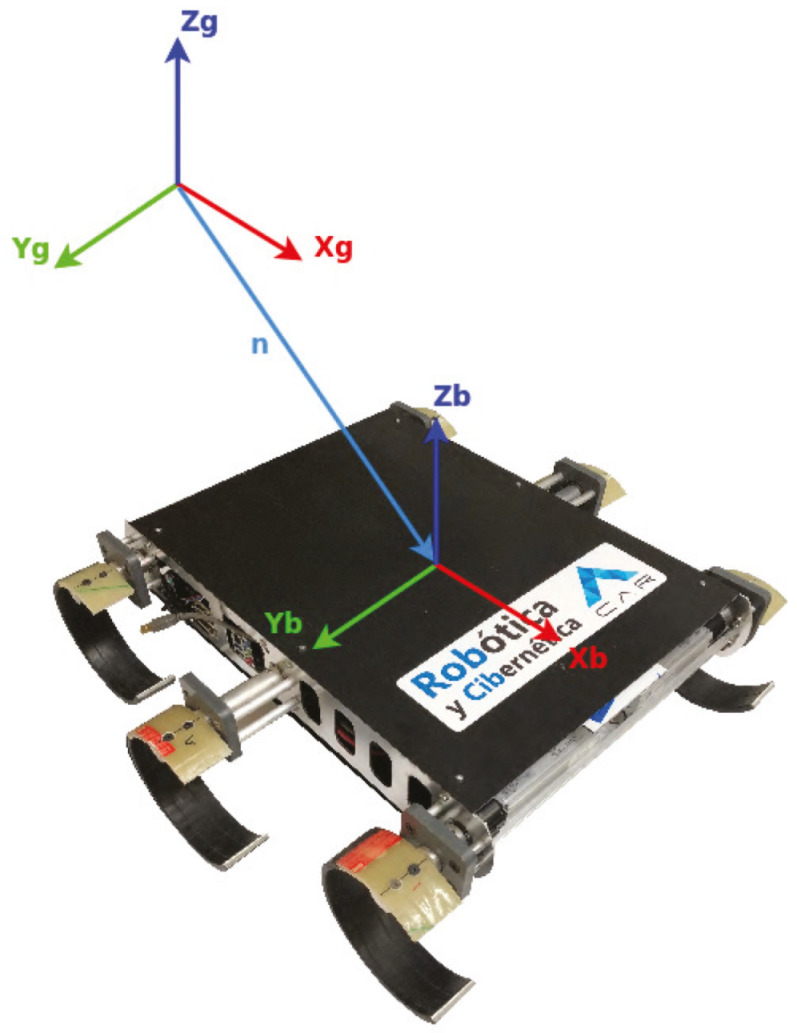
Global (G) and Body-fixed (B) Coordinate Frames for CLHeRo V2.5.

The convention for the reference system is showed on the Figure 3. The assignment for the reference system is accord with the SNAME (Society of Naval Architects & Marine Engineers [51]) notation. The dimensions of the robot are required for generate its physical model, the most relevant characteristics are the dimensions and mass. These characteristics and a comparison with some of the robots mentioned in the introduction is shown in Table 2.

The CLHeRo platform can be configured with 2 different computers. If the user wants only to teleoperate the robot and send video via streaming, the best option is a Raspberry PI 3 B+. The reason is because for teleoperating the robot, all the gaits pattern algorithms can be run in a processor with low specifications. Moreover, the RPI 3 B+ consumes about 400 mA of current at 5.0 VDC (which is about 2 watts), that increases the power autonomy of the robot greatly.

The second option is intended to proved to the robot high level tasks, like autonomous navigation, 3D reconstruction or Visual SLAM. As these tasks requires a high computational cost and send all the data collected to via wireless to compute it in the ground station and send back the information is impracticable, we decided to install a computer with a powerful processor. Unfortunately this configuration reduces drastically the power autonomy of the robot. In the Table 3 a description of the components of the computer are list.

Both, the RPI 3 B+ and the computer with x64 bits architecture must have installed the same Debian version and ROS distribution (Kinetic and Melodic have been tested successfully) in order the code generate for controlling the motors can be used indistinctly.

The computer controls the robot’s gait and other behaviors, gathers and logs sensory information from various parts of the system, and communicates with the control station. This communication is maintained via either and internal wireless card connected to the computer or an external wireless solution for wide ranges as payload.

**Figure 4:**
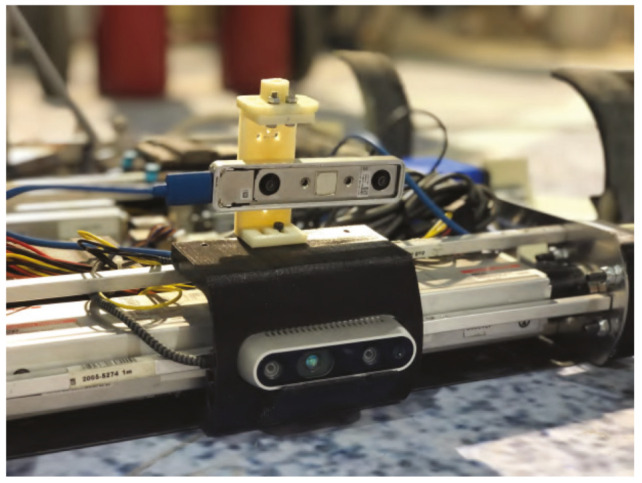
Intel D435 and T265 cameras.

In addition to the sensors used in the motor control and battery management, there is a SparkFun 9DoF Razor (SEN-14001) which combines a SAMD21 microprocessor with an MPU-9250 9DoF (9 Degrees of Freedom) sensor, placed at the center of the chassis, that provides inertial sensing of the robot. The 9DoF Razor’s MPU-9250 features three 3-axis sensors an accelerometer, gyroscope and magnetometer that give it the ability to sense linear acceleration, angular rotation velocity and magnetic field vectors. The onboard microprocessor, Atmel’s SAMD21, is an Arduino-compatible, 32-bit ARM Cortex-M0+ microcontroller also featured on the Arduino Zero and SAMD21 Mini Breakout boards. It also has an official ROS package (http://wiki.ros.org/razor_imu_9dof). In the front of the robot there is a RealSense D435, which is an active stereo depth camera that uses Intel’s custom ASIC, the Intel RealSense VisionProcessor D4, to conduct a custom variant of the Semi Global Matching algorithm to compute the depth. It also has an optional infrared (IR) projector that assists in improving the depth accuracy by projecting a non-visible static IR pattern when the scene’s texture is low, can get up to 848 × 480 @90 frames per second. Upper the Intel RealSense D435 there is an Intel RealSense T265 tracking camera (see Figure 4) which outputs the current pose (position and orientation) 200 time per second. The camera has two fish eye lenses with combined 1635 FOV and BMI055 IMU Sensor on board. Visual Inertial Odometry from Intel is running on board.

The MCD control unit includes the EPOS controllers, also from Maxon. This motor controller comes preprogrammed with a variety of control modes [52] (profile position, profile velocity, homing mode, interpolated position, position, velocity, current, master encoder and step direction). While using a controller like this, one saves the time and effort needed to develop the gait control modes.

The EPOS includes a 32 bits@60 MHz microprocessor for managing all the parameters with 256 KB of free memory in case the user wants to store a program. This microprocessor closes a low-level feedback internally at a rate of 10 KHz; this high speed loop permits motor current targets to be reached and accurately. In addition to control loops, these family of controllers handle the sinusoidal commutation for the brushless motors, provide the sensor feedback for position, velocity, current and temperature of the motor.

These units can be controlled with two different communication protocols: RS-232 at 115200 bauds and CanOpen at 1 MB/s.

For using the CanOpen protocol is necessary to acquire one of the recommended PC-CAN interface cards. We use the IXXAT USB-to-CAN v2. Once, all the motors were wired and configured, it works perfectly with the example *cpp* program (tested under Ubuntu 16.04). Unfortunately ROS framework does not accept this protocol, even there are some developments like ROS_canopen and Kacanopen, they have not implemented the functions for controlling the MCD units with all their functionalities. Moreover, the ROS package “epos_hardware” developed by the RIVeR-Lab [53] was tested. This package was programmed to control the Maxon EPOS 2 controllers via USB, but with a modification in the code made by Jimmy Da Silva [54], the serial communication was available. The connection with the motors was successfully, but when we try to write or read from the controllers, there was a big delay in the communications. This delay caused that the execution of the robot’s control failed.

Therefore, to solve this problem, we developed a new ROS package to manage the communications with the Maxon EPOS and implemented a mixed communication network. This option creates an internally CanOpen network between all the motors, assigning an identifier to each motor. Then, the CanOpen master unit established an external communication with the computer via the RS-232 protocol. This master node manages the information for all the motors and sent it to the target unit.

## 3. Robot Kinematic

As was explained in the introduction, the CLHeRo robot walks with a GP called alternating tripod, which is inspired in some insects like beetles or cockroaches. From a engineering point of view, it can be described as a differential robot.

**Figure 5:**
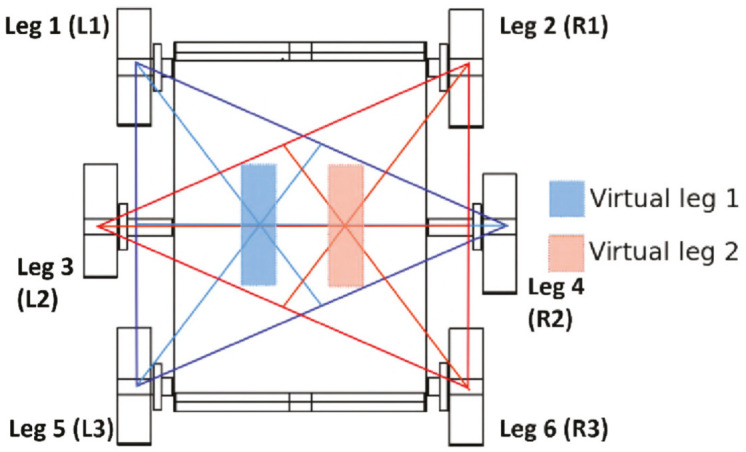
CLHeRo 2.5: Tripods and virtual legs.

To achieve the differential mode, the robot combines its six legs as two virtual legs (Figure 5). Each virtual leg includes the front and the back leg from a side, and the middle one from the opposite side. Therefore, the tripod 1 (T1) is formed by the frontal left leg (L1), the back left leg (L3) and the middle right leg (R2), on the other hand, the tripod 2 (T2) is formed by the frontal right leg (R1), the back right leg (R3) and the middle left leg (L2).

The cyclic sequence described by the alternating tripod established that at every step one tripod is in the aerial phase and the other in the ground phase. At the aerial phase no reaction forces are present, while in the ground phase each leg experiment the following forces:

- The ground reaction forces vector, which is break down into two components, the X and the Z.
- This is assumed because the robot only moves in the sagittal plane. Together with gravitational force.
- The moment generate by the motor.

The reaction forces have to take in consideration the flexible properties of the legs, because the radius of the leg varies together with the motor rotation. So it is necessary to add the formal spring expression $\left(-K_{i}\left(l_{i}-l_{0}\right)\right)$.

**Figure 6:**
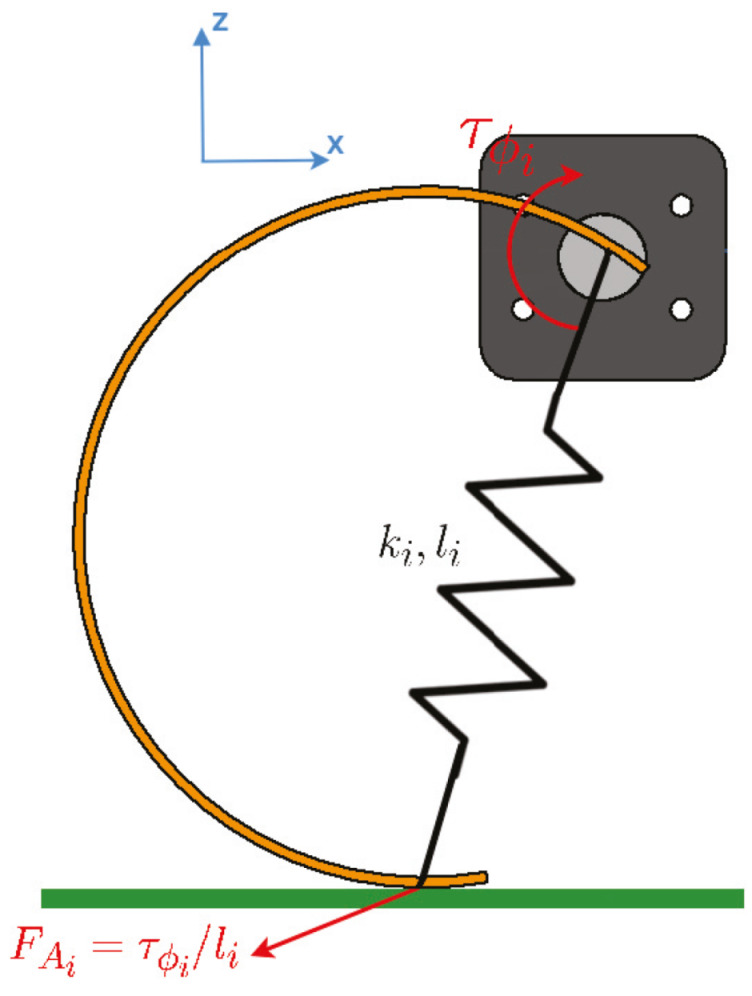
Leg forces on the $XZ^{B}$ plane.

From Figure 6, the moment of the motor is $\tau_{\phi_{i}}$ and the reaction forces is $F_{A_{i}}=\frac{\tau_{\phi_{i}}}{l_{i}}$, where $\tau_{\phi }$ is the torque generated by the *i* motor in the DoF ϕ and $l_{i}$ represents the length of the leg at every moment because of the passive flexion produced due the flexible properties of the “C” leg. Therefore, when a leg *i* is in contact with the ground experiments a reaction force ($F_{A_{i}}$) directly proportional to the torque generated by the motor *i* and the length of the leg *i*.

For a better comprehension of the kinematic model for the CLHeRo, an exhaustive study is published in [49].

## 4. Odometry Estimation

As was explained in the introduction, to achieve some applications with the robot is necessary to require the information of the actuators of the robot. In this section the authors explain how is implemented the odometry for the CLHeRo robot.

### Legs Odometry

As was mentioned in the introduction, calculate the odometry for the “C” legs robots is not as easy as in wheeled robots. Various are the factors that have to be take into consideration:

- The rotation axis is not locate at the center of the leg.
- The trajectory described by the leg is a cycloid.
- The leg presents elastic properties.
- At every cycle, the leg rotates without being in contact with the ground.

#### Odometry Model

**Figure 7:**
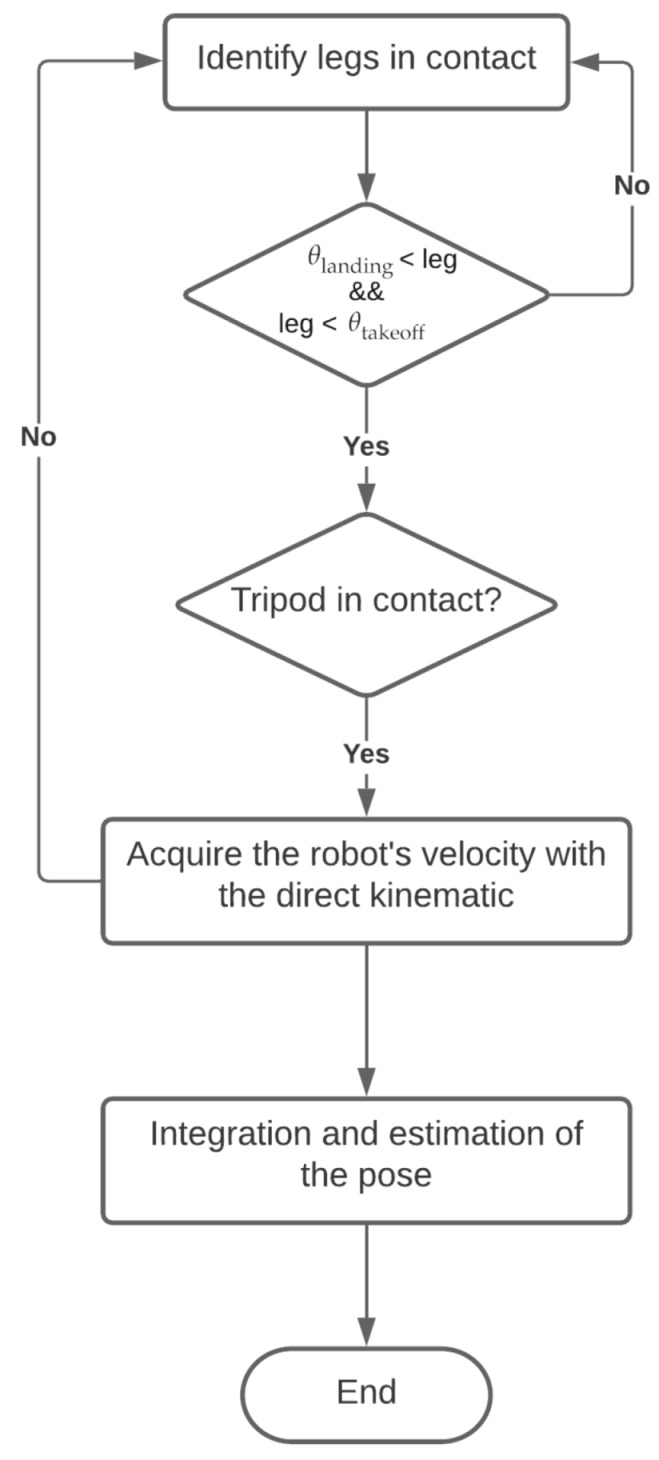
Odometry model steps.

The mathematical model for the odometry has been developed considering future improvements (modifications on the CLHeRo chassis) and the adaptability of the model to any “C” legs robot (can be adapted to “C” legs with different width, radius, elasticity or material). The model follows the steps shown in Figure 7.

Moreover, for the mathematical model the authors have made the following assumptions:

- The robot’s movement is planar
- The legs roll without sliding or skidding.

Identify the legs in contact with the ground:

In contrast with the mobile robots with wheels that always are in contact with the ground, the legged robots need to identify which of these are in the position to transmit the effort.

This identification proceeds in 2 steps: a filter that chooses those legs which position can be in contact with the ground and a final identification through a weighting function that selects the legs which extension to the ground is higher.

**Figure 8:**
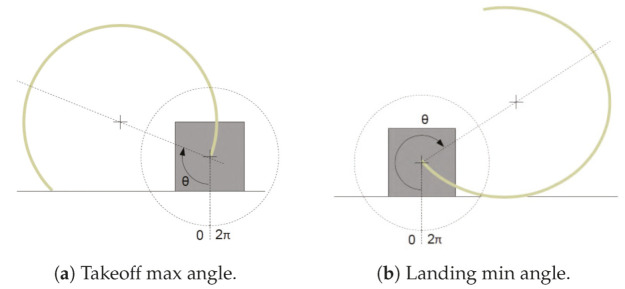
Limit angles for aerial movements.

The first step filters those legs that have a configuration valid to be in contact with the ground. Due to the CLHeRo’s geometry, exists a range of rotation for the legs where it cannot touch the ground, this points are called, takeoff max angle ($\theta_{takeoff}^{max}$) and landing min angle ($\theta_{landing}^{min}$). The value of each is obtained with the geometric relations from the previous work [48], and for the CLHeRo V2.5 with a leg with 160 mm of diameter are shown in the Equation (1). Figure 8 shows the limits. Both represent the boundary between the aerial and ground movements, being this the origin of their names.

(1) $$\theta_{\mathrm{takeoff}}^{max}=103.7287^{{}^{\circ}};\;\theta_{\mathrm{landing}}^{min}=241.954^{{}^{\circ}}$$

From this, the condition for the filter is shown in Equation (2).

(2) $$\mathrm{Leg}\;\mathrm{in}\;\mathrm{possible}\;\mathrm{ground}\;\mathrm{position}⟺\left\{\begin{array}{c} \theta \in \left[0,103.7287\right]{}^{{}^{\circ}}\\ \theta \in \left[241.954,360\right]{}^{{}^{\circ}} \end{array}\right.$$

Due to the configuration of the hexapods, the minimum number of legs that configure a stable situation is with 3, a tripod. Any configuration with a number of legs less than 3 and more than 0 is considered as an unstable position, that either the robot cannot move or if it did it would be uncontrolled, thus, in that case it is considered that the robot remains halt, see Equation (3).

(3) $$\mathrm{If}\;\mathrm{legs}\;\mathrm{in}\;\mathrm{possible}\;\mathrm{ground}\;\mathrm{position}<3⟹{\bar{v}}_{robot}=0$$

If the number of legs is equal or superior to 3, the identification will continue. Only the tripod with the 3 legs closest to the maximum elevation position of the robot is considered to realize the traction with the ground. The maximum elevation occurs in the position 0 or 2π.

**Figure 9:**
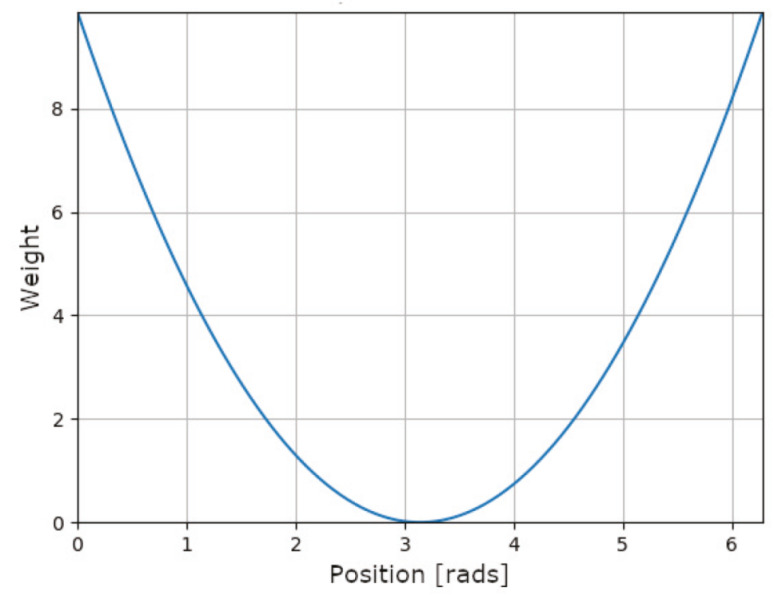
Weighting function for elevation.

The elevation of each leg is assessed by a weighting function that contributes with a score proportional to itself. This weighting function (Equation (4)) corresponds to a second degree polynomial function with a double root at π, point of lower elevation. Figure 9 represents this function in the definition interval of the legs position. A leg is perpendicular to the ground at 0 rads or 2π rads, as a leg get nearer to this position, the weighting function scores higher that leg. Otherwise, at π rads the leg is at the highest position; therefore, the score obtained is zero.

(4) $$f_{pond}\left(\theta \right)={\left(\theta -\pi \right)}^{2}$$

Once the elevation score is obtained, these are in order of highest to lowest ratings with a quicksort algorithm [55] and the 3 legs with highest score are chosen. Then, the state of these 3 legs will be used to obtain the velocity of the robot.

When the legs that are in traction with the ground have been identified, the velocity of the robot is calculated from the state of the legs with the kinematic of the CLHeRo. The direct kinematic implemented is based in the same model of other mobile robots [56,57,58]. Grouping the legs into two tripods the CLHeRo presents a kinematic similar to a differential-drive robot, as Siegwart explains, where each tripod/wheel contributes to the motion.

Using this method, the velocity of the robot in a leg can be defined as:

(5) $${\bar{v}}_{leg}={\bar{v}}_{robot}+\bar{\omega }\times {\bar{r}}_{rp}$$

**Figure 10:**
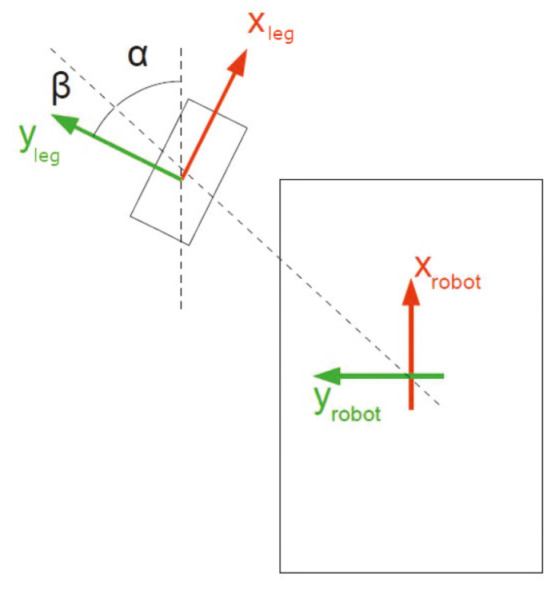
Representation of parameters used in the kinematics for a leg.

Now, expressing the forward and rotation velocity of the robot from the reference system of the leg, the general expression for the direct kinematic is obtained (see Equation (6)). This expression is similar to other systems that can be found in the literature for a model with a unique leg [56].

(6) $$\left[\begin{array}{c} v_{leg,i}\\ 0 \end{array}\right]=\left[\begin{array}{ccc} s(\alpha_{i}+\beta_{i}) & -c(\alpha_{i}+\beta_{i}) & -d_{i}\cdot c\left(\beta_{i}\right)\\ c(\alpha_{i}+\beta_{i}) & s(\alpha_{i}+\beta_{i}) & d_{i}\cdot s\left(\beta_{i}\right) \end{array}\right]\left[\begin{array}{c} v_{rx}\\ v_{ry}\\ \theta_{r} \end{array}\right]$$

where α is the angular position of the leg with respect to the center of the robot. β is the angle that forms the *Y* axis with the line that joins the center of the robot with the center of the leg and *d* is the distance between the center of the robot and the leg. Figure 10 shows a diagram with these parameters.

The velocity of the leg only presents the term of forward velocity in the *X* axis, because one of the assumptions of the model is that the legs neither slip nor skid.

So, the velocity of the robot expressed from a fixed reference system is obtained by rotation matrices. Therefore, the direct kinematic for a *i* leg can be expressed as (Equation (7)):

(7) $$\left[\begin{array}{c} v_{leg,i}\\ 0 \end{array}\right]={\bar{\bar{R}}}_{i}\left[\begin{array}{c} v_{x}\\ v_{y}\\ \dot{\theta_{r}} \end{array}\right]⟹{\bar{v}}_{p,i}={\bar{\bar{R}}}_{i}\cdot \bar{v}$$

This represents the general model of the direct kinematic for mobile robots, nevertheless, to particularize for the CLHeRo is necessary to specify the velocity of the legs and solve the values of α and β.

The legs of the CLHeRo do not present any DoF in the direction of the leg, consequently, they are always attached with the same orientation with respect to the chassis of the robot. This peculiarity causes that the α and β parameters present fixed values for each one of the legs.

(8) $$\mathrm{For}\;\mathrm{every}\;i\;\mathrm{leg}⟹\alpha_{i}+\beta_{i}=\frac{\pi }{2}$$

This demonstration makes that the ${\bar{\bar{R}}}_{i}$ matrix can be simplified in the following form (Equation (9)):

(9) $$\left.\begin{array}{c} s\left(\alpha_{i}+\beta_{i}\right)=s\left(\frac{\pi }{2}\right)=1\\ c\left(\alpha_{i}+\beta_{i}\right)=c\left(\frac{\pi }{2}\right)=0 \end{array}\right\}\Rightarrow {\bar{\bar{R}}}_{i}$$

$${\bar{\bar{R}}}_{i}=\left[\begin{array}{ccc} R1 & R2 & R3 \end{array}\right]$$

$$R1=\left[\begin{array}{ccc} 1 & 0 & -d\cdot c(\beta_{i})\\ 0 & 1 & d\cdot s(\beta_{i}) \end{array}\right];R2=\left[\begin{array}{ccc} c(\theta ) & s(\theta ) & 0\\ -s(\theta ) & c(\theta ) & 0\\ 0 & 0 & 1 \end{array}\right];R3=\left[\begin{array}{ccc} c(\theta ) & s(\theta ) & -d\cdot c(\beta_{i})\\ -s(\theta ) & c(\theta ) & d\cdot s(\beta_{i}) \end{array}\right]$$

To obtain the velocity of the legs is necessary draw on their kinematic, which has been presented by the authors in previous works ([33,47,48]). The kinematic of the legs corresponds to a cycloid; therefore, the velocity can be defined as:

(10) $${\bar{v}}_{p,i}=\left[\begin{array}{c} v_{p}\\ 0 \end{array}\right]=\left[\begin{array}{c} R\cdot \left[\dot{\varphi }-\dot{\varphi }\cdot cos\left(\varphi \right)\right]\\ 0 \end{array}\right]$$

where φ is the position angle of the cycloid and *R* is the radius of the leg. The algorithm, also includes a mathematical lineal approximation to the nominal length of the radius of the length, this lineal approximation is obtained after the results of several simulations with finite elements analysis of the “C” legs and allows to implement the effective radius at every step and avoid calculating the mesh elements and the requirement of high computational capabilities to estimate the pose at every step. To reference the kinematics with the same angle, is possible to make the next change of variable.

(11) $$\varphi =\pi +\theta ⟹\dot{\varphi }=\dot{\theta }$$

Finally, the expression for the velocity for any leg is:

(12) $${\bar{v}}_{p,i}=\left[\begin{array}{c} v_{p}\\ 0 \end{array}\right]=\left[\begin{array}{c} R\cdot \left[\dot{\theta }-\dot{\theta }\cdot cos\left(\pi +\theta \right)\right]\\ 0 \end{array}\right]$$

At this point, is necessary to remember the assumptions made by the authors: the movement of the robot has only be considered in the plane, this does not take into account the displacement in the *Z* axis. And the legs have an ideal rolling without sliding or skidding, which makes the velocity in the *Y* axis zero.

Nevertheless, so far it has only applied the kinematic for a leg, giving rise to a undetermined system a priori. To apply it to the rest of the legs, which receive the subscript i,j,k is enough with extend the same definition.

(13) $$\left[\begin{array}{c} {\bar{v}}_{p,i}\\ {\bar{v}}_{p,j}\\ {\bar{v}}_{p,k} \end{array}\right]=\left[\begin{array}{c} {\bar{\bar{R}}}_{i}\\ {\bar{\bar{R}}}_{j}\\ {\bar{\bar{R}}}_{k} \end{array}\right]\cdot \left[\begin{array}{c} v_{x}\\ v_{y}\\ \dot{\theta } \end{array}\right]⟹{\bar{v}}_{p}=\bar{\bar{R}}\cdot \bar{v}$$

Since there are a greater number of restrictions to that of DoF, it may result in an incompatible system. So with minimum squares the minimum error solution can be obtained, that will be taken as an estimate of the robot’s speed.

Pose integration and estimation:

Once we have estimate the velocity of the robot with the direct kinematic, the estimation of the pose is done with the last position and the numerical integration.

(14) $${\bar{v}}_{robot}={\left[{\bar{\bar{R}}}^{t}\cdot \bar{\bar{R}}\right]}^{-1}{\bar{\bar{R}}}^{t}\cdot {\bar{v}}_{p}$$

The final expression for the pose obtained is taken as an estimate of the pose resulting from the odometry of the legs.

## 5. Tests and Validation

The objective with the tests is to verify two objectives:

- The validation of the proposed model for the odometry algorithm
- The analysis of fusing the odometry information with more sensors and compare if the localization is more precise.

The simulation tests were run with the Gazebo simulator version 7 and the model detailed in the C-Legs ROS metapackage [49]. On the other hand, the real tests can be distinguished between indoor and outdoor tests. The indoor tests were monitored with a ground truth system to validate the measurement of the different sensors, while the outdoor tests uses the onboard sensors of the robot and manual measure tools.

### 5.1. Simulation Tests

Test environment description: As was mentioned above, the software used for the simulations has been Gazebo simulator version 7, which is the default simulator for ROS Kinetic. For the tests Gazebo was updated to the version 7.14. However, everything has been test and is possible to run it with Ubuntu 18.04, ROS Melodic and Gazebo 9.0.

The implemented model for the simulation faithfully reproduces the physically characteristics of the robot and the behavior of the robot’s actuator.

The empty default world from Gazebo was used to carry out the tests, because to analyze the gait patterns is not necessary to implement any special scenario.

Test conditions: For analyzing the forward displacement 10 tests were performed. The robot and the control program were configured for the fiberglass legs with 160 mm of diameter, a ground sweep angle of 60 degrees, a rotation velocity for the legs of 1 rad/s at the ground phase and to complete 10 steps.

After each test, the final position error and the mean squared error were analyzed to verify the accuracy of the algorithm.

**Figure 11:**
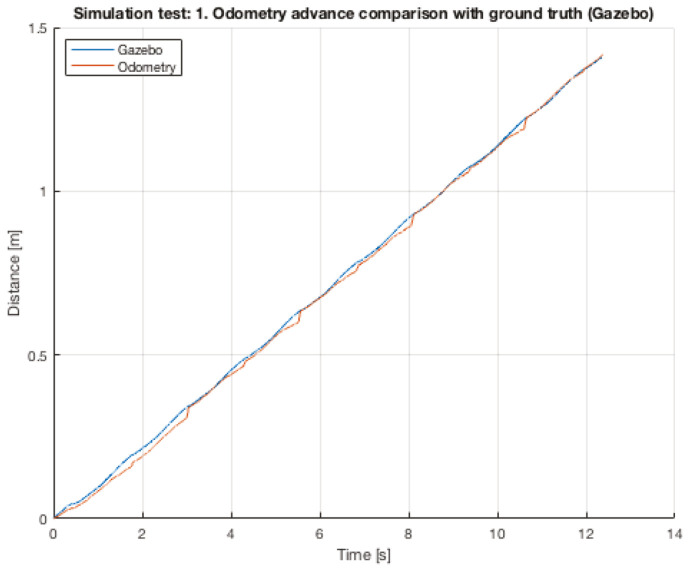
Gazebo test #1.

Conclusions and discussion:

Is important to point, that one of the reasons why the results vary is, largely due, to the manually initialization of all the ROS nodes and they stop. So, the human factor has an important effect on the results. However, despite that, the variations in each tests are very small. In the Table 4, the mean squared error and the maximum error for each test are listed. The error is measured between the pose of the robot given by Gazebo and the estimation calculated by the odometry algorithm. Figure 11 shows the results for the test #1. Analyzing in detail the graph, it is possible to observe that the measures from Gazebo have a continues slope, while the odometry algorithm present the particular jumps at every step, for example at time = 3 [s] or time = 8 [s].

The objective of these simulations were to analyze if the odometry algorithm was enough accurate as the other sensors mounted on the CLHeRo, for that reason, only the forward displacement was analyzed in the tests.

The mean of all the mean squared errors and the mean of the maximum errors are very small, see results below:

$$\mathrm{Mean}\;\mathrm{MSE}=0.0015\;m^{2}$$

MeanMaximumError=0.0954m

If we do not take in consideration the results from the test 7, which presents a result outside of the mode, the new value of the mean for the maximum errors is reduced to 0.0069 m. This means that for all the tests were the robot has walk a mean distance of 3.257 m, the odometry algorithm has an estimation error less than 7 cm, in other words, 2.14% of the traveled distance. This is a very accurate result considering that the algorithm uses an approximate model of the leg that makes a lineal approximation of the flexible characteristics of the leg. Or an error less than the 10% of the total body length of the robot. So, the authors consider that the results obtained from the odometry algorithm have sufficient precision to use it in the real robot.

The graphs for the simulation results of the tests, the simulation tests dataset and the Matlab scripts can be downloaded from this repository (https://github.com/grafoteka/clhero_pose_tests).

### 5.2. Indoor Real Tests

**Figure 12:**
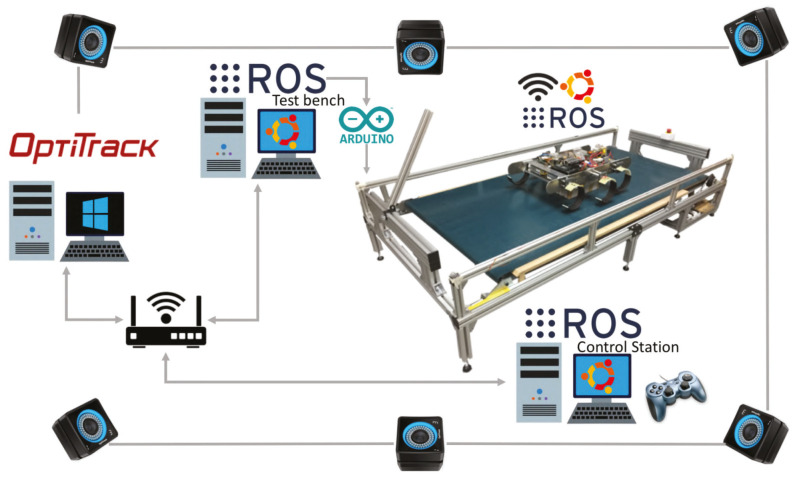
Schema of all the system for the indoor tests with the test bench.

**Figure 13:**
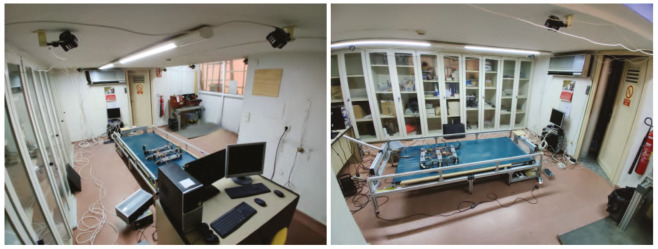
Test room for the odometry tests.

Test environment description: To carry out the indoor tests, two scenarios were set up for this purpose. The first one, include a test bench developed in previous works [59] and a Optitrack motion capture system to obtain the pose of the robot. This test bench has been designed to analyze the forward displacement of the robot and analyze different gait patterns and configurations (rotation speed, attack angles, …). The test bench includes a computer that uses the Optitrack system information to calculate the difference between the pose of the robot and the center of the test bench and thereby regulate the speed of it. Figure 12 shows the schema of all the system for the real tests and Figure 13 shows the test room once it was operational. In this room, a total of 6 cameras of the Optitrack system were installed together with a computer exclusively dedicated to running the Optitrack control software (Tracking Tools). The effective volume that is covered with the cameras was focused on the test bench since it would be the area where the robot would perform the tests.

**Figure 14:**
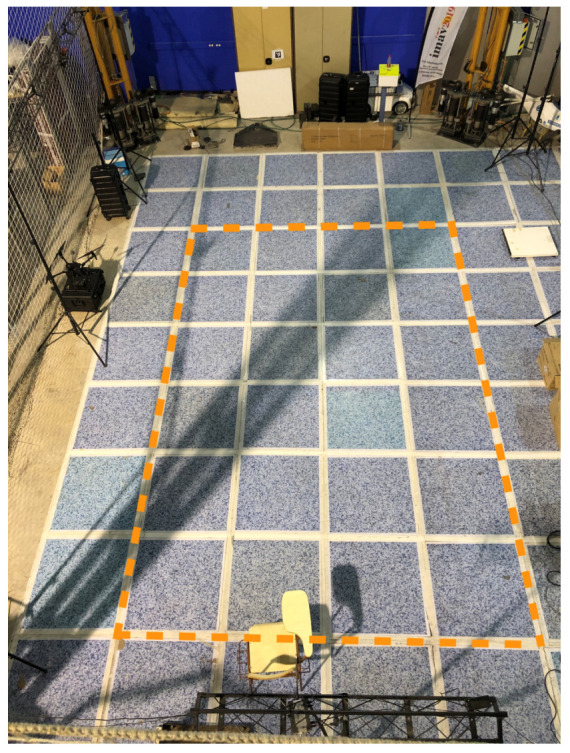
Indoor tests scenario 2.

The second indoor scenario area is bigger than the first one and has been conceived to analyze more complex maneuvers like turning or complete a circuit. The effective work area for this scenario is 8 × 6 m. For covering this area, two more cameras were necessary to be installed, so a Optitrack system with 8 cameras was configured. As in the first indoor scenario, one computer is exclusively necessary to run the Optitrack control software and publish the global pose of the robot. Figure 14 shows the second test area once it was operational.

#### 5.2.1. Indoor Tests—Scenario 1

**Figure 15:**
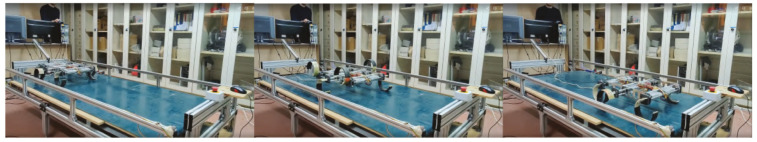
CLHeRo forward tests—scenario 1.

Test conditions: The tests carried out in the test bench (Figure 15) aimed to validate the results obtained in the Gazebo simulations. The real tests consisted on a set of 10 tests walking forward on the test bench. In order to reproduce the conditions of the simulation, the configuration of the parameters of the robot were the same as in the simulations, see Table 5.

In order to prevent a result influenced by the human factor, the teleoperation of the CLHeRo was forbidden and a *ROS C++ script* was created, so in each test the execution orders were send always with the same time-stamp. To reproduce or execute the same tests, the reader can download the codes and rosbags available in this repository (https://github.com/grafoteka/clhero_test_bench).

In this tests, the set up of the robot was without its batteries, instead of that, it was supplied with a cable that was also used as an umbilical cord, like in some real search and rescue tasks. In the other hand, the communications between the robot and the control station were untethered, with Wi-Fi protocol at 2.4 GHz.

Results and discussion: As was explained before, the objective with this tests is to validate if the odometry algorithm is enough accurate to use it in the real robot and can be used as an input data for fusion sensor. Like in the simulation study, in the test bench tests, only the forward displacement of the robot has been taken into consideration.

**Figure 16:**
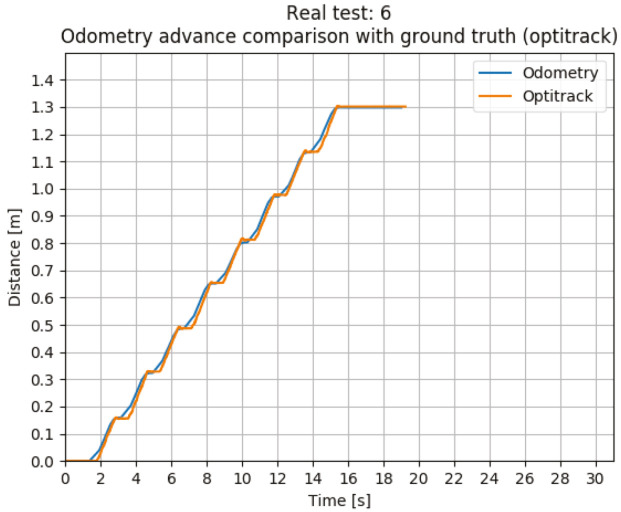
Real test #1.

In the Table 6, the mean squared error and the maximum error for each test are listed. The error is measured between the pose of the robot given by Optitrack system and the estimation calculated by the odometry algorithm. Figure 16 shows the results for the real test #6.

For all the tests the robot has walk a mean distance of 1.259 m, which is equal to 8 steps with a ground sweep angle of 60 degrees. The odometry algorithm has an estimation error of 2.5 cm at the final position, the mean of the odometry values is 1.269 m and the mean for the Optitrack measures is 1.244 m, in other words, 1.97% error of the traveled distance.

$$\mathrm{Mean}\;\mathrm{MSE}=0.0055\;m^{2}$$

MeanMaximumError=0.1096m

The tests show that the results obtained are even better than the obtained in the simulations. But some facts have to be pointed, the distance traveled by the robot is less in the real tests than in the simulations, so tests with a longer distance traveled are required to evaluate if the error is constant or increases with the distance. Second, some physical parameters or coefficients cannot be modeled in the simulation, so it could be another point to take into consideration.

#### 5.2.2. Indoor Tests—Scenario 2

**Figure 17:**
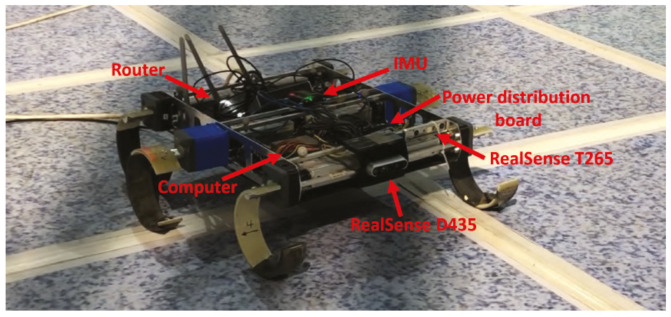
Components installed on the CLHeRo for the tests.

Test conditions: For the indoor scenario 2, three different tests were performed: Walking straight, turn in place and complete a circuit. The first one, walking straight, can be considered as an extension of the test in the indoor scenario 1. However, this time the analysis will include all the sensors installed in the robotic platform (IMU, RealSense D435 and T265). On one side, the IMU is used together with the odometry algorithm and the ROS package *Robot pose EKF* to estimate the pose of the robot. It uses an extended Kalman filter with a 6D model (3D position and 3D orientation) to combine the input measurements. On the other side, the RealSense D435 is used with the algorithm ORB-SLAM2 [60] which is a SLAM solution to compute in realtime the camera trajectory and a sparse 3D reconstruction. It is able to detect loops and relocalize the camera in realtime. Finally, the RealSense T265 uses the Intel tracking software to calculate the position and orientation of the robot. Figure 17 shows all the components for this test and the following.

Walking straight test: The walking straight test can be subdivided into two different tests. The first one consists in 5 trials to analyze the final localization of the robot and the error measurement from each sensor respect to the ground truth system. These analyzes include the XY trajectory of the robot and three individual studies of the displacement in the three axis respect to the time (*X*, *Y*, *Z*). The *XY* trajectory is used to recreate the path followed by the robot in the trial and get the final error in the coordinates (*X*, *Y*). The individual studies of each variable is used to find some periodic behaviors or disturbance in the sensor measurements. For this trials the robot configuration is the same as in the indoor scenario 1 (see Table 5).

Table 7 resumes the final errors for each trial and sensor. The “Odometry” tag results are the EKF values with the input of the odometry algorithm and the IMU sensor, but is called with that name, for a better comprehension.

**Figure 18:**
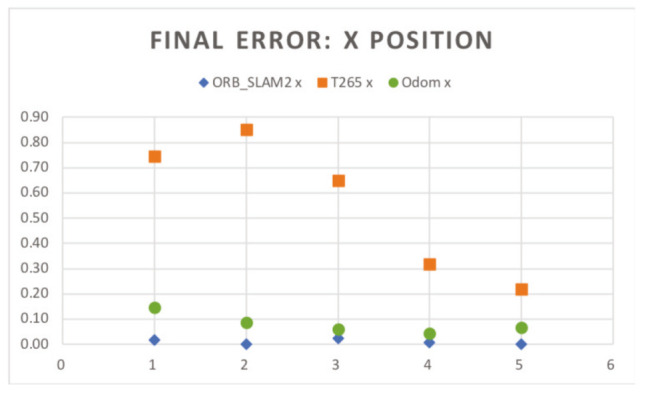
Indoor tests scenario 2. Test 1. Trial 1. Final error X position.

**Figure 19:**
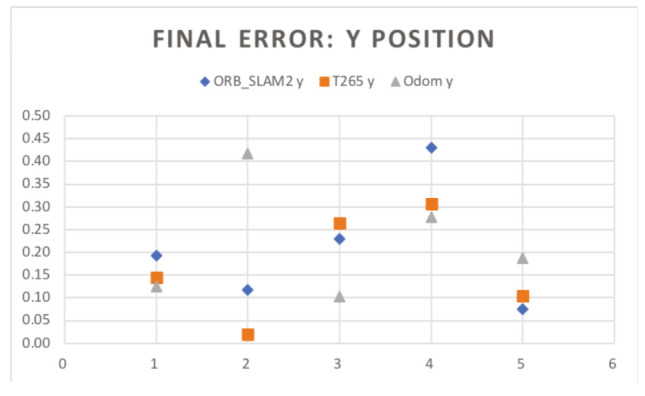
Indoor tests scenario 2. Test 1. Trial 1. Final error Y position.

**Figure 20:**
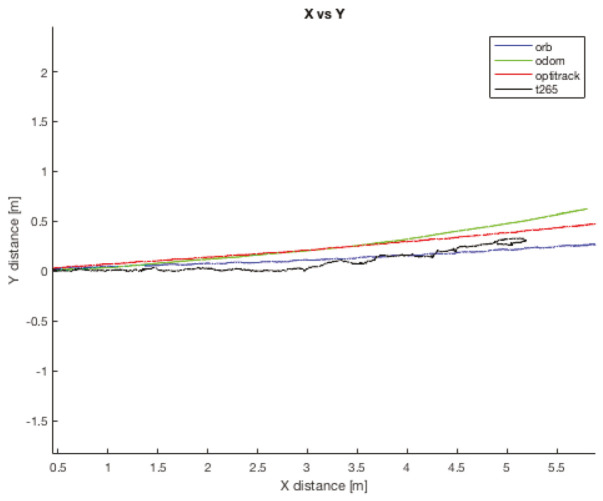
Indoor tests scenario 2. Test 1, trial 1. Final error XY position.

In the Figure 18 and Figure 19, the results of the final *X* and *Y* errors are shown. The T265 presents much worse errors than the ORB SLAM2 and EKF algorithms, even though *Intel* specifies that the algorithm of the T265 uses an EKF algorithm together with the onboard IMU sensor of the camera. The magnitude of the error for the trials 1 and 2 is even bigger than the length of the body of the robot. However, if we study, for example, the XY graph of the trial 1 (see Figure 20), it shows that the T265 presents a more erratic and not as smooth path as the ORB SLAM2 path. After some more tests, the reason why the T265 presents this error in the measurements is because the Intel’s Visual SLAM requires more features in the scene than the ORB SLAM2 algorithm. When the amount of features that are present in the scene increases, this error is reduced.

So, the error measure in the final X position has acceptable results, specially for the EKF and ORB SLAM algorithms, however, none of the three methods presents a perfect estimation for the displacement in the Y axis. However, the errors measured are smaller than the half of the width of the length of the chassis of the robot, which can be marked as an acceptable result.

**Figure 21:**
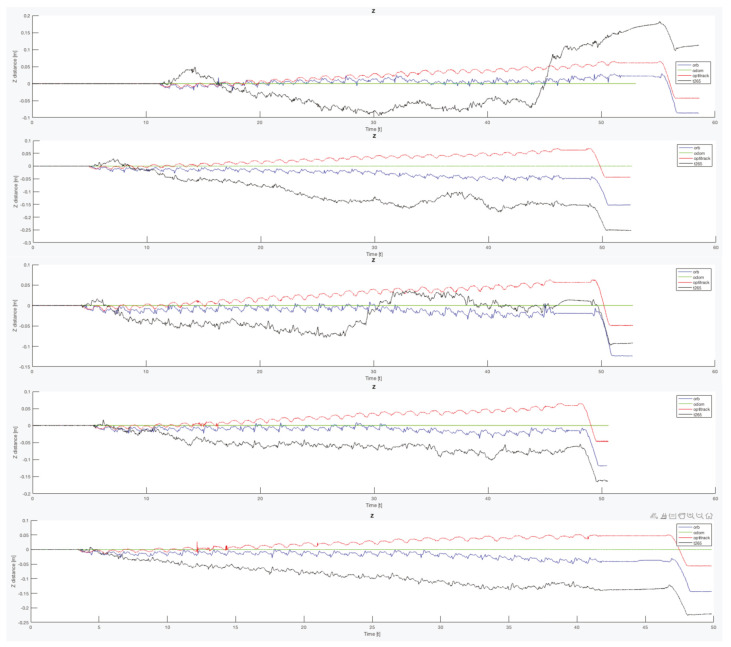
Indoor tests scenario 2. Test 1, trials 1–5. Error in Z position.

Another important point is that, for all the trials, the T265 presents a worse estimation of the robot position in the Z axis. While the measure should be between 0.0 [m] and −0.1 [m] in some trial, the T265 exceeds the +0.2 [m], which is a difference as big as go up one step. Figure 21 resumes the Z measures from the first test, the black line is the T265 camera.

The second test consisted in 3 different trials, and each one was repeated twice. Moreover, in each one of the three trials the ground sweep angle was modified (30°, 45° and 60°). The objective of this second test is to analyze if different gait patterns configurations help to achieve a better pose estimation and try to find a better configuration to solve the errors showed by the T265 camera. For the six tests, the CLHeRo was programmed to walk for a period of sixteen strides and not to achieve a certain distance, because the distance travelled in each step is directly correlated with the ground sweep angle. The ground rotation speed parameter has kept constant at 1 rads/s. Moreover, for this test, more EKF filters were configured. This new EKF filters have been configured in pairs of sensors (Odometry + IMU, Odometry + ORB SLAM2, IMU + ORB SLAM2), so now the study can also indicate if a couple of sensors make a great difference in the pose estimation of the robot. Moreover, the EKF filter for this test include three sensors measurements (Odometry, IMU and ORB SLAM2). To verify the conclusion from the previous test, where the T265 was not very accurate, some objects were included in the scene, so the T265 can extract more features in each frame.

**Figure 22:**
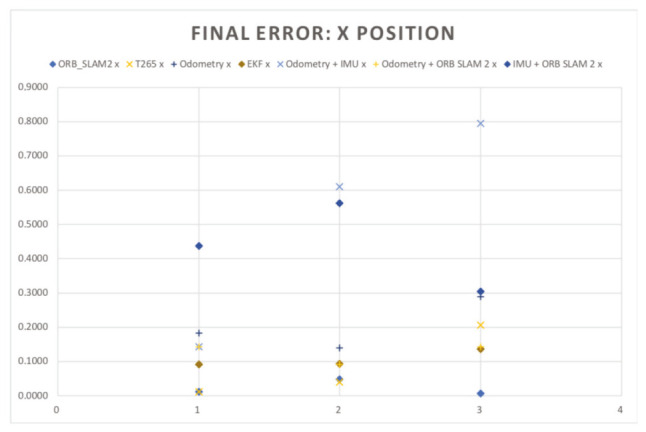
Indoor tests scenario 2. Test 1. Trials 1–5. Final error X position.

**Figure 23:**
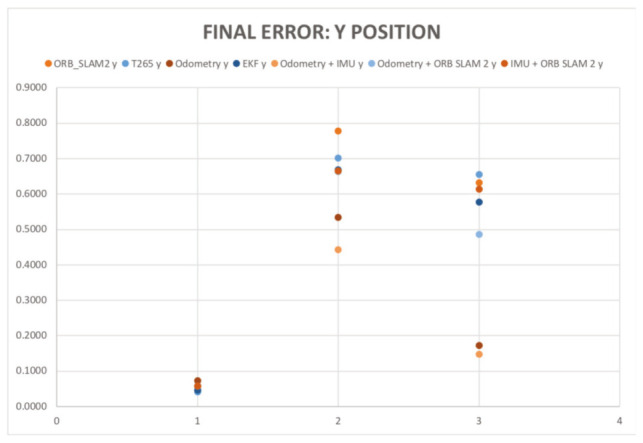
Indoor tests scenario 2. Test 1. Trials 1–5. Final error Y position.

Table 8 resumes the final mean errors for each trial, sensor and EKF combination. In the Figure 22 in and Figure 23 the results of the final X and Y errors are shown.

The first thing that the reader can notice from the results is that the final error in X position for the T265 has decreased considerably (in some cases more than 0.6 m which is an improve of the 85%). In the 6 trials the raw measurements of all the sensors is below than 0.2 m of error and in the majority of cases under 0.1 m, which can be considered as a very precise results. The worst results are the combination of the EKFs that combines the IMU with another sensor (odometry or camera), it could be caused by the oscillations and forces that the robot suffers at every step. Decreasing the rotation speed of the motors can help to reduce this negative effect.

The final Y error position has also been significantly improved. The gait pattern with the ground sweep angle of 30 degrees has proved to be a very precise gait pattern in order to estimate the pose of the robot in both axis (X and Y). While in the two other configurations (45 degrees and 60 degrees) a major oscillation of the robot’s body provokes that the cameras have several problems to perform a better estimation and therefore the EKF that includes the odometry and the IMU presents the better results.

**Figure 24:**
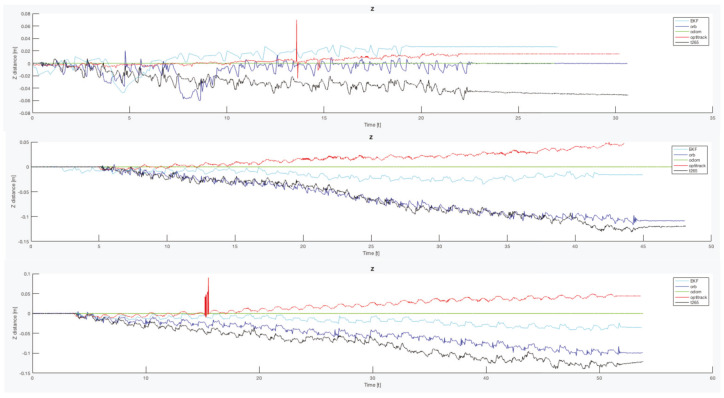
Indoor tests scenario 2. Test 1, trials 1–5. Final error Z position.

Finally, to complete this analysis, is necessary to compare the oscillation in the Z axis (Figure 24). As happened with the X and Y final errors, the Z position error with the legs angle configured as 30 degrees presents the better estimation, but only for the ORB SLAM2 algorithm. For the other two configurations, both visual estimators presents a negative derivative, but with the combination of the EKF algorithm with the three inputs (odometry, IMU, ORB SLAM2) this negative error can be solved and the Z error position is almost null.

Turn in place: This type of movement has been the second to be implemented in the CLHeRo. Having this two different actions (walking straight and turning in place) for moving the robot, allows to achieve more complex tasks in a future, e.g., path following or autonomous navigation.

In order to have a more detailed study for this test, it was divided in five trials, where only the ground sweep angle was modified. The first trial begin with a configuration for walking straight, 60 degrees (1.05 rads). After that, in the next trials the angle was reduced: 45,30,22.5,11.25 degrees (0.79,0.52,0.39,0.2 rads). However, in all the trials the ground rotation velocity was kept constant: 0.1 rads/s.

Moreover, in this test and in the futures one, to have a more detailed analysis the EKF combinations with couples of sensors have been configured, as in the previous test. And taking in consideration some lessons learned from the previous test, different objects were included in the scene in order to have more features and get a better tracking.

The first trial, with the 60 degrees configuration failed, the robot was only able to do some steps and then was necessary to do some recovery maneuvers and resynchronize the legs to be able to continue turning. After several tries, this trial was considered as null due to the lack of torque of the motors.

The second trial, with 45 degrees (0.79 rads) configuration, was also repeated several times, and each trial consisted in two and a half turns. The extra turns in each trial were done because both cameras were lost. So, closing the loop was intended to relocalize the robot after each complete turn. However, this only was useful to correct the ending position of the turn, during the rest of the turn there was a translational error that could not be reduced.

The configuration with 30 degrees (0.52 rads) was the third trial. In this case, the same strategy as in the previous configuration was repeated, turn more than one turn in order to close the loop and relocate the robot.

For the last two trials, 22.5 and 11.25 degrees (0.39 and 0.2 rads) configuration, only one turn was executed, because in these configurations, the oscillation in the *Y* and *Z* are less aggressive than in the previous cases.

**Figure 25:**
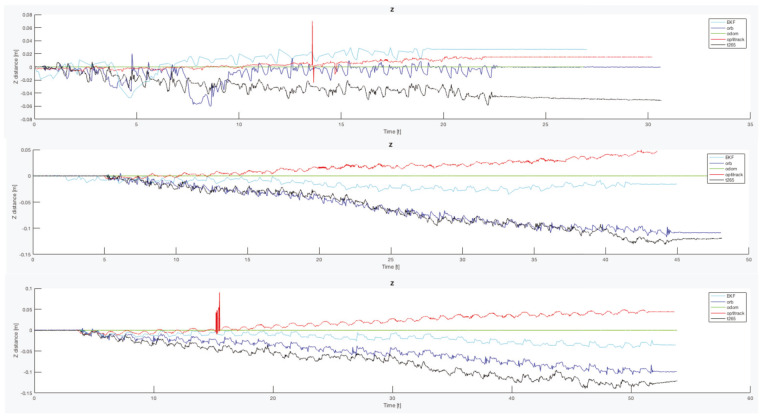
Indoor tests scenario 2. Test 1, trials 1–5. Final error XY position.

Figure 25 shows the tracked XY position for the trials 2–5. It is important to notice that both localization methods based on images present the same and constant error. It draws the attention that for all the trials, the visual error have more or less the same magnitude and presents the shape of a circumference. In the case that the robot needs to realize one complete turn, there should not be any problem because the XY localization is correct (less than 10 cm of error, which is an acceptable value). Nevertheless if the robot only turns a portion of a complete turn, a translational error appears in the robot localization, it can get a maximum value of the length of the robot’s body for 180 degrees or half of the body length for 90 degrees or 270 degrees, Table 9 and Table 10 show in detail this error.

For its part, the odometry algorithm, except for the last trial, presents a good estimation of the position of the robot, with only a few centimeters of error. This error is provoke because the algorithm takes in consideration the maneuver of turn in place as an ideal turn with no friction or drift, but this is not true, because of the morphology of the robot and its properties to rotate the legs, is necessary some drift to be able to turn.

With the final results of the previous tests, the authors can confirm that the odometry algorithm presents a good accuracy to estimate the pose of the robot. Some reasons to accept the algorithm as valid are that the estimation error is not increased with the distance and the estimation is better when it is compared with other sensors like the T265 that is highly accepted for autonomous navigation with ground and aerial robots.

Now, analyzing the EKF results, in one hand, the *IMU + VO* combination does not improve the estimation, so this configuration can be rejected. In the other hand, the complete EKF and the *odometry + VO* reduces the effect of the translational error in a range about the 50% (see Table 11). And finally the *odometry + IMU* shows an excellent result, drawing a perfect turn in place movement.

One possible reason why the visual odometry sensors a loosing the localization of the robot is due to the complex movement of the turn. In each step, the robot rotates, but also some vertical displacement is registered. This vertical displacement is bigger as the ground sweep angle is increased, table shows the average vertical displacement at every step depending on the angle.

Circuit: This test combine the movements studied in the last two previous explained tests. This test can be used as a first approach to achieve autonomous navigation for robots with “C” shape legs.

The circuit consisted of traversing a rectangle with approximate dimensions of 3 × 2 m. A total of four trials were done for this test, and all of them were teleoperated, so each one differ a little bit in the path generated. The important facts for the study is not to do always the same path, but are: first, that the path covered presents the minimum possible error between the Optitrack system and the information from the sensors. Second, the final position must be as close as possible to the physical final position.

**Figure 26:**
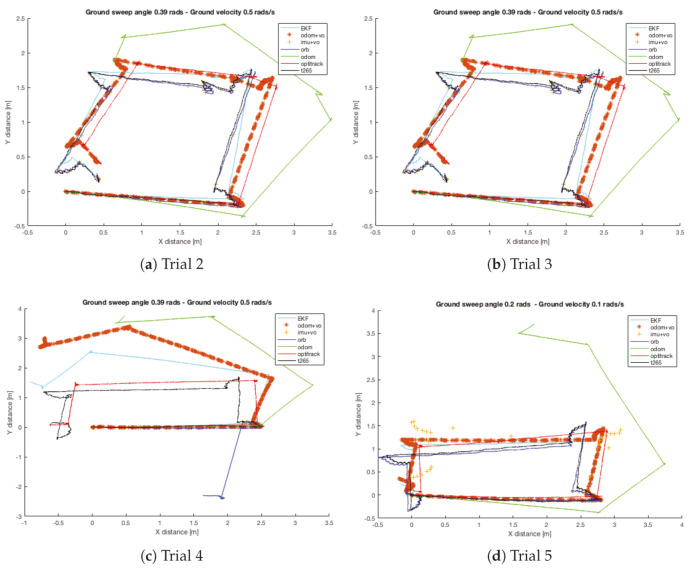
Indoor tests scenario 3. Trials 2–5. Error in XY position.

Figure 26 shows the trajectory followed by each trial. The three first trials were done with a turning configuration of: ground sweep angle 0.39 rads and a ground rotation velocity of 0.5 rads/s. In the third trial, the ORB SLAM2 algorithm lost the robot localization. For that reason, a more conservative setting was selected (ground sweep angle of 0.2 rads and ground rotation velocity of 0.1 rads/s). However, for both cases, the walking straight configuration was 60 degrees and 1.0 rads/s. Table 12 resumes this information.

Analyzing the results, some conclusions can be done from a first sight: the odometry algorithm has failed in all the trials at every turn action. So, the odometry algorithm needs to be improved to have a better accuracy when alternating different types of movement. The second conclusion is that the translational error described in by the visual odometry sensors in the *turn in place test* is also present in the turns of this test. Eventhough the translational error exists, there is not a problem with the orientation because the walking straight path is parallel to the path described by the Optitrack system.

Now, studying the final error position error, there is not a great difference between the different sensor to identify which one is the best one. The *ORB SLAM2* algorithm and the *T265* camera present very similar results. For the *X* position error, both sensors are giving a precision under the 4 cm of error. Meanwhile, for the *Y* position, the *T265* average error is 0.11 m and for the *ORB SLAM2* (excluding the trial 3) the average error is 0.159 m.

Attending now to the EKF combinations, only the *odom+vo* configuration helps to reduce the final error in both axis (*X*, *Y*). Although for the trials *2* and *4* there is not a real benefit or improve. In the opposite side, the *imu+vo* configuration have a worse result in all the trials than the Visual SLAM methods by themselves. Finally, the complete EKF presents a similar performance as the *odom+vo*, but in this case, it is able to correct the predicted trajectory along the path and is very similar to the one followed by the robot. Table 13 resumes all the final errors from this test.

### 5.3. Outdoor Real Tests

Finally, to complete this work, some outdoor tests were performed in order to analyze the precision of the algorithms and sensor in real conditions. Because of the characteristics of the outdoor scenarios is not possible to have the Optitrack system, but a conventional measurement system, which can give a precision of millimeters is used.

Two of the three tests explained in the previous section were adapted to the outdoor scenario, the walking straight and the circuit. Moreover, each test was carried out in two different types of terrain: asphalt and meadow. The first one offers a high coefficient of friction in addition to be a flat surface. The second one is an uneven terrain with remnants of cut vegetation on the surface, this can provoke that the legs drift.

#### 5.3.1. Walking Straight

**Figure 27:**
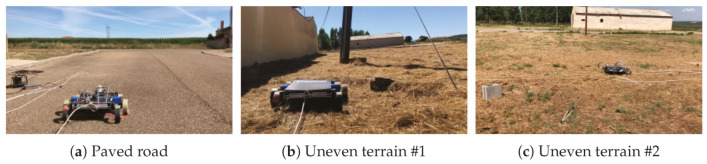
Real tests scenarios.

Test conditions: The walking straight test was carried out in three different places: one paved road and two different uneven terrains (see Figure 27).

The paved road presents a high coefficient of friction (friction coefficient between rubber and dry asphalt = 0.9 [61]) between the asphalt and the rubber that covers the legs of the robot, so the rotation of the motors can be transform into forward displacement without losses.

In the two uneven localizations, the remnants of vegetation cause that the legs can present some loss of traction on some steps. Moreover, the ups and downs of the terrain induce that for a total forward displacement, more steps will be required than from a flat surface.

For both types of terrain, the robot was configured with the same parameters, which are established as the default configuration for walking straight in alternating tripod, and are resumed in Table 5.

Each trial had a different final position because of the characteristics of the terrain, Table 14 resumes the final position measure for each trial. It is important to notice, that for the uneven terrain is not possible to have a certain measure of the real displacement in the Z axis due to the lack of a ground truth system. While for the asphalted surface the authors assume that is completed flat, so the initial Z height is equal to the final Z height.

**Figure 28:**
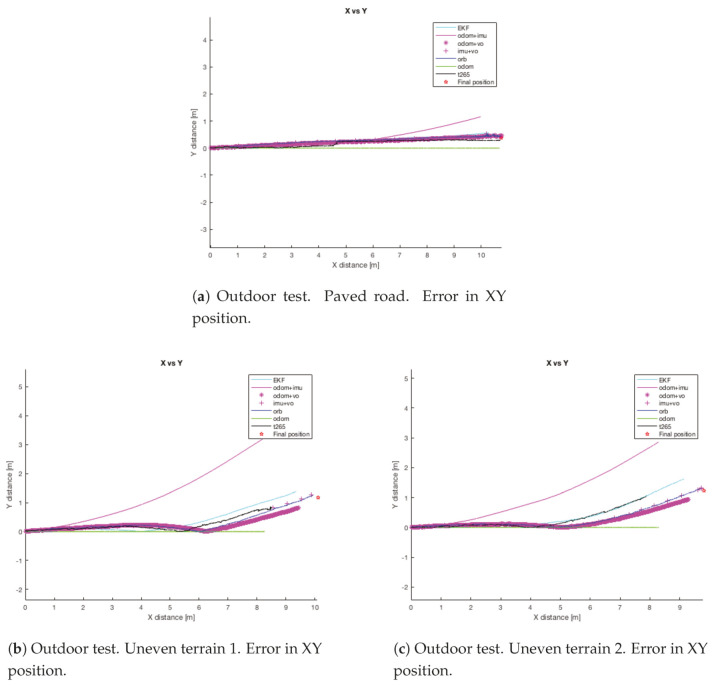
Outdoor test #1.

Results and discussion: Table 15 resumes the mean error for the final position in each trial and Figure 28 shows the path followed by the robot in each one.

**Figure 29:**
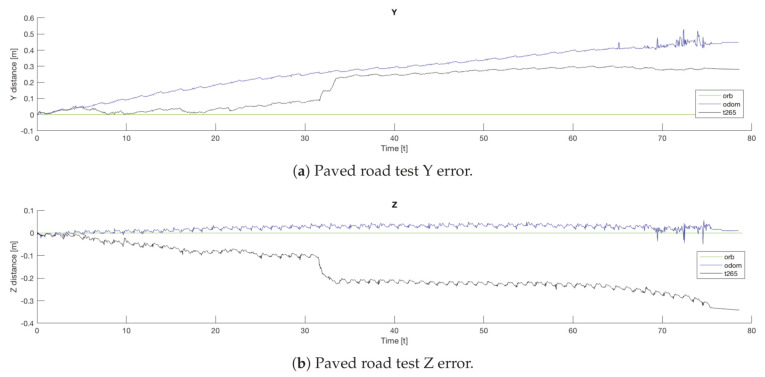
Outdoor test #1.

For the paved surface all the sensors and EKF combinations, with the exception of the *odom+IMU* configuration, present a good final position estimation. The majority of the methods have a final *X* error less than the *0.5%* of the distance traveled. In the other hand, for the final *Y* position error, both visual slam methods present an error around the *20%* of the final position. However, the *ORB SLAM2* algorithm reflects the real trajectory of the robot, while the *T265* corrects the position after detecting an approximate error of *20* cm., see Figure 29a. Moreover, the full *EKF* configuration presents a higher error than the *ORB SLAM2* because it has to correct the error from the odometry algorithm estimation, which it does not add any displacement in the *Y* axis.

If now we attend to the *Z* graph of the same trial (Figure 29b), the *T265* shows a negative drift that is never corrected and with each step it is increased. This error could be caused because the camera should need more features in each frame, as happened in the previous indoor experiments.

**Figure 30:**
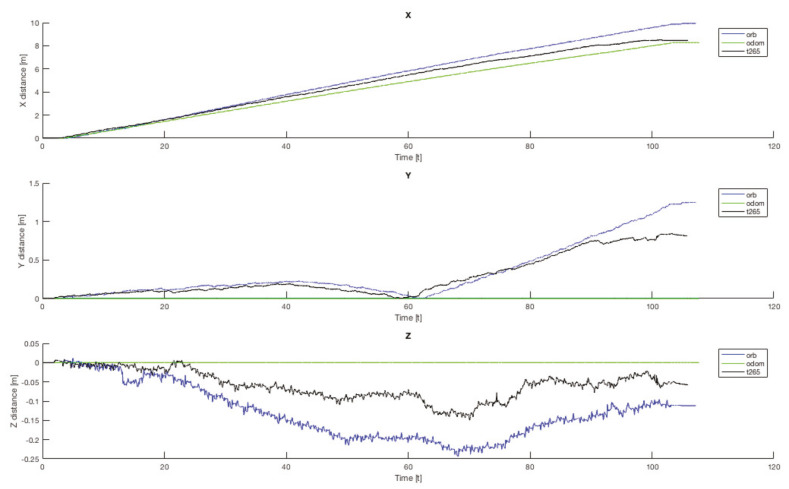
Outdoor uneven terrain #1. Walking straight test. Absolute errors.

The first trial on uneven terrain shows an error similar to the detected on the asphalt, the *IMU* shows a big drift from the beginning of the trial. Another point is that the *T265* camera stopped tracking the position almost at the end of the experiment (see Figure 28b). Moreover, the odometry algorithm presents a large error in the final *X* position. So, the best estimation is done by the *ORB SLAM2* algorithm. In this trial, the *Z* displacement cannot be measure, but *ORB SLAM2* and *T265* presents a similar curve, and that is a good sign. Figure 30 shows the absolute error in each axis for this test.

For the second uneven terrain trial (Figure 28c), again the *IMU* shows a drift that is not related with the path followed by the robot. It may be possible that the *IMU* algorithm cannot filter the perturbations provoked by the displacement on the uneven terrain. Moreover, due to this behavior of the *IMU*, the full *EKF* combination shows a deviation from the path followed by the robot. The analysis of the *X* distance traveled by robot reveal that one more time the *ORB SLAM2* algorithm together with the *RealSense 435* is more precise than the other two methods included in the robot (*odometry and RealSense T265*). However, for this trial, the *odometry algorithm* has less error than the *T265*, although this error can be considered as bad estimation (18.36% of the total distance). On the other hand, the *EKFs* that include the visual odometry as input have corrected the error of the odometry (75.56%) and the full *EKF* (91.43%). Attending now to the *Y* error, both visual sensors presents a good estimation, the *T265* even better than the *D435*, despite it has a worse result for the *X* position.

**Figure 31:**
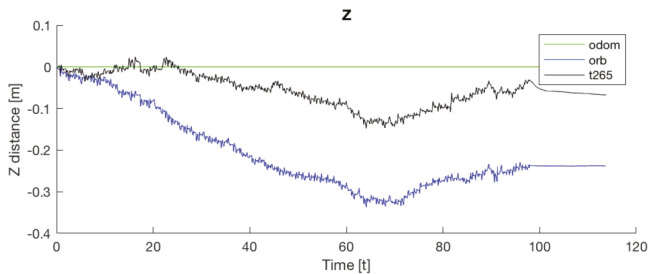
Outdoor test 1. Uneven terrain #2. Absolute Z error.

Like in the previous test on uneven terrain, both visual sensors have a parallel trajectory on the *Z* axis but with a difference of *20* cm, nevertheless there is not option to identify which one is more accuracy, see Figure 31.

Finally, it is possible to confirm that for walking on a paved road the odometry algorithm and the visual slam methods present very accurate results during the path and at the final position. However, when the robot has to walk through uneven terrain the odometry algorithm presents a worse estimation, always considering that it has traveled less distance. Moreover, the combination with the *IMU* presents a bad result because the *IMU* cannot filter the disturbance generated by the steps and that are increased with the obstacles on the surface.

#### 5.3.2. Circuit

Finally, to complete this work, the last test carried out was to complete a circuit, but only on the paved road and on the uneven terrain #2. The experiments on the asphalt were repeated twice and in both occasions the robot was able to complete it. However, on the uneven terrain the trial was repeated five times and only in one occasion the robot could achieved the end point successfully. The reason why the robot could not success all the test is provoke by the characteristics of the terrain and the maximum torque that the motors can achieved. As the terrain is not flat, in some steps all the legs are not in contact with the ground; therefore, some motors have to increase the torque generated to move the robot to the next step. Sometimes it was possible, but when the torque required is too high, the motor’s protection for over current is activated and does not allow to move the motor. This could be solved by acquiring a motor with a higher torque, but it will decreased the maximum travel velocity.

**Figure 32:**
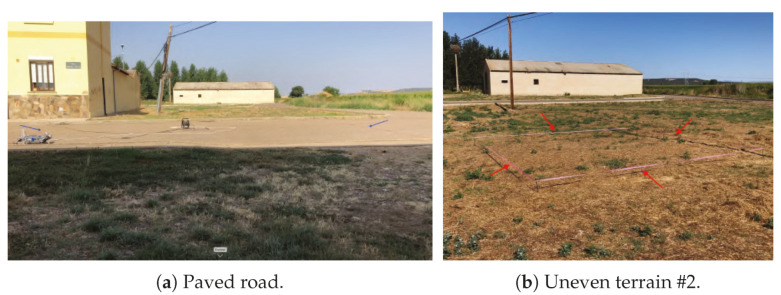
Outdoor test #2.

The circuit on the paved road had a maximum width of 9 m (the width of the road), so the circuit describes a square of 9 × 9 m, see Figure 32a. Thus, the trajectory of the robot will be inscribed in this square. While the circuit on the uneven terrain is a square of 4 × 4 m (Figure 32b).

As happened in the indoor experiments, the *IMU* is not detecting correctly the turning action and points that the robot has turn less than 90 degrees in each corner. Therefore, with that large measurement error the estimation of the full *EKF* is not valid either.

**Figure 33:**
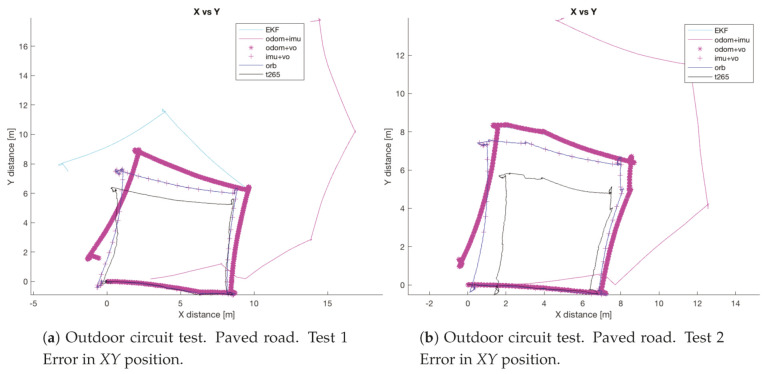
Outdoor circuit test on paved road.

**Figure 34:**
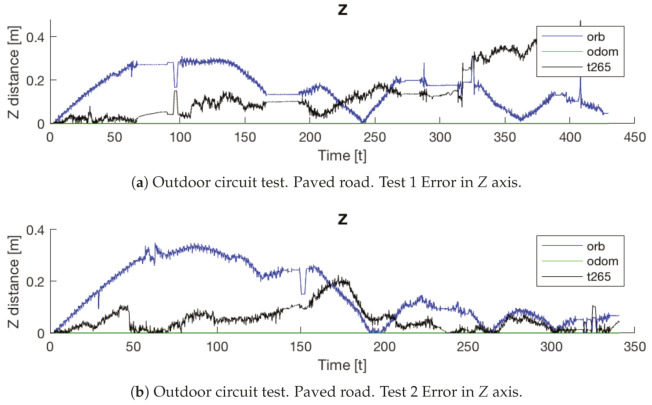
Outdoor circuit test on paved road. Error in *Z* axis.

In both experiments on the paved road (Figure 33), the *T265* camera describes a path shorter than the showed by the *ORB SLAM2* algorithm and followed by the real robot, but is able to recognize that has closed the loop and correct the final position. For the paved road, the second trial obtained a better pose correction from the full *EKF*, very similar to the *odom + vo* configuration, but in both cases the best estimation is with the *ORB SLAM2* algorithm. Despite, the measures in the *Z* axis are not valid (see Figure 34) because its values are out of the range that the robot can achieve, furthermore it presents high variations.

**Figure 35:**
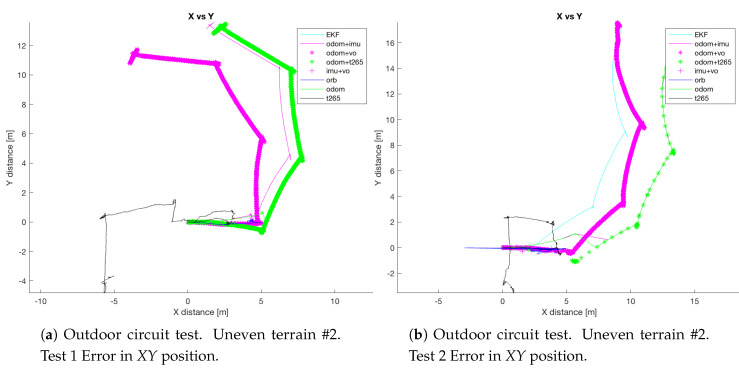

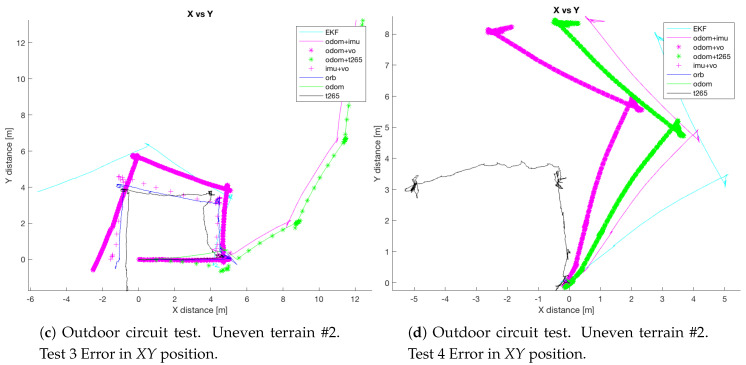
Outdoor circuit test on paved road.

The circuit on the uneven terrain was more difficult to complete for the robot, in three of the four experiments, the result was unexpected. The *ORB SLAM2* algorithm stopped tracking the position because it lost the references, even the *T265* not always gave a good estimation, it never stopped tracking the position, while the *odom + IMU* still showing a big error. The best pose estimation was on the third test (see Figure 35).

Due to the fail of the *ORB SLAM2* algorithm and the drift from the *IMU* sensor, all the *EKFs* combinations that include one of these two sensors, or both, shows a result that is not representative of the path followed by the robot. So, for the circuit test on the uneven terrain, only the results from the third test are valid for this analysis.

Like in al the previous circuit tests, the *odometry* algorithm and the *IMU* are not giving a good estimation; therefore, the full *EKF* combination deteriorates the pose given by the *ORB SLAM2* algorithm. To try to correct this, for the combinations with the *ORB SLAM2* algorithm the *EKF* configuration was modified and more weight was given to the data received from the algorithm.

Attending now, only to the results from the *T265* and the *ORB SLAM2* (that one more time are giving the best results), the *T265* describes a curve on the right side of the circuit, after the first turn, and it is not correct, it should be a straight line. Moreover, the path show by this sensor indicates that the robot continue walking straight more than one meter after reach the initial *Y* point. Therefore the *ORB SLAM2* algorithm shows a more accurate result.

## 6. Conclusions

The authors have present in this work a complete study that includes different maneuvers, scenarios and configuration parameters for estimate the pose in C-legs robots with a novel odometry algorithm and the conjunction with other sensors to evaluate how useful it is. In addition, this work not only analyze the behavior of the robot for a few steps in a straight path.

In contrast to previous studies like [35,36], this study is focus in the alternating tripod gait and not in the analysis with dynamics gaits like jogging.

With the results obtained, the authors can confirm that the algorithm have a precision similar to the visual odometry methods and, in scenarios with few visual characteristics can have better results. Moreover, can be used in robots that do not have a high performance computer because the algorithm does not required high computational costs as the *ORB SLAM2* algorithm. And in some other conditions, specially in the turn in place maneuvers the odometry algorithm can help to improve the estimation of the visual and *IMU* sensors.

In Section 4, the authors defined the algorithm for a “C” leg with a initial diameter of 160 mm, however, due to the intrinsic flexible properties of the leg and the variations during the displacement of the CLHeRo, this length varies continuously. Calculating the exactly length of the leg in real time with FEA methods is not possible due to the actual limitations of computer calculating; therefore, after the simulation tests and the indoor tests—scenario 1, a linear approximation of the average compression of the leg was approached. This approximation was generated for the physical characteristics of the CLHeRo v2.5, the “C” legs properties and the velocity and acceleration profile specified for the rest of the tests. Moreover, this “new” length of the leg, was introduced as a parameter in the odometry algorithm which can be interpreted as a correction factor for the nominal diameter of the leg. It is important to point, that for an optimal implementation in other platforms, is necessary to do some tests with different parameters and analyze the results to obtain the adequate factor for the new platform.

Despite, there are some limitations and problems that have to be take in consideration, which will be described below.

### 6.1. Problems and Limitations

#### 6.1.1. Motors Data Update Frequency

Due to the communication problems, involving the Maxon interface and the ROS protocols, which are problems unrelated to the focus of this work, the frequency to obtain the parameters of the motor (current position, velocity, and torque) is very slow (8 Hz). Considering that the odometry is obtained with a callback message, the intervals for the numeric integration are high. If the publish frequency would be higher, like in the Gazebo simulations (100 Hz), the odometry results could be more accurate by reducing update times.

#### 6.1.2. Legs Elasticity

One of the characteristics of the “C” legs robots is the elasticity of its legs, which provides to the robot the ability to perform unique actions, nevertheless this implies the deformation of the legs when they are in contact with the ground.

To solve this situation, for the fiberglass legs, a elasticity coefficient has been included in the equation. For this work, using a value of 0.9125 has demonstrate to be valid. This value has been calculated taking in consideration the robot standing with 3 legs, its mass and the compression parameter of the fiberglass legs (k=8166 N/m).

A good proposal could be to test the robot with different legs (varying the its diameters and elasticity) and walking configurations (angles and speeds) to create a database. So the algorithm could be adapted perfectly to different C-legs robots.

#### 6.1.3. IMU

The *IMU* installed in the robot is frequently used in different robotics platforms like autonomous ground vehicles [62] or underwater vehicles [63]; and for other applications like 3D reconstruction with visual odometry [64]. Nevertheless, in none of these previous works the *IMU* presented an erratic performance as in the study presented here. Analyzing the displacement in the Z axis (see Figure 30, Figure 31 and Figure 34) the reader can observe that the *IMU* is reading constantly variances in the measurements, and therefore adding a drift in the estimation of the pose causing loss of confidence in the sensor.

### 6.2. Computational Cost

The *ORB SLAM2* algorithm used for the calculating the robot localization with the RealSense D435, demands high compute capabilities, using this algorithm with the RPi 3 is not possible because the processor does not have enough capabilities. For this option, the best configuration can be to only use the Odometry algorithm proposed by the authors together with the RealSense T265, which computes all the tracking system in the camera and only sends a message with the position and orientation of the robot. Nevertheless, using the *ORB SLAM2* algorithm offers a very precise and robust localization, even for outdoor environments. And with the results of the tests it needs less features in the scene than the RealSense T265.

Having in consideration this problems and limitations, the authors are aware that the implementation of the odometry model presented in this study for fusing the information with other sensors can improve the pose estimation of a “C” legs robot. Moreover, the results obtained after the tests can help to understand that this kind of robot has peculiar characteristics and the maneuver of turning can provoke to lose the estimation of the pose, specially because the visual odometry systems are used when the turning action only presents a rotation in the Z axis without any perturbation in the other two axis.

The next steps to improve the pose estimation of the robot, specially for the indoor scenarios can be the use of other sensors like *LIDARs* and other techniques like *SLAM* so the error can be reduced and for outdoor scenarios the use of a *GPS* or *A-GPS* be very helpful.

Another future implementation will be the detection and recognition of steps to automate autonomous climbing of stairs; the authors can identify at this moment the steps of a stair using a velodyne, but the objective is to be able of do the detection with the RealSense D435 to avoid adding more sensors. Moreover, the sequence for stair climbing has been successfully tested in simulations using finite state machines with an algorithm developed with Matlab/Simulink software. If all this implementation can be done in the real platform, the CLHeRo could be able to autonomously locate, navigate and create a map in buildings with several floors.

Finally, the authors wants to remark that this work presented is all available in their repository (https://www.github.com/grafoteka/clhero_common) and can be tested following the steps explained in the “Test and Validation” section and the results of the tests carried out (https://github.com/grafoteka/clhero_pose_tests).

## Figures and Tables

**Table 1:** Degrees of freedom (DoFs) for different configurations of legged robots.

Configuration	DoF/Extremity	Extremities	Total DoFs
Biped	6	2	12
Quadruped	3–6	4	12–24
Hexapod	3–6	6	18–36
Octopod	3–6	8	24–48

**Table 2:** Physical properties of the robots. All measures in mm, except the total weight (kg).

Attribute	Body Height	Overall Width	Body Length	Leg to Leg Spacing	Ground Clearance	Inverted Ground cl.	Leg Diameter	Total Weight
RHex [7]	139	390	500	200	115	95	175	8.9
Rugged [18]	148	465	623	235	106	106	195	15
EduBOT [23]	108	340	360	155	90	N/A	117	3.6
X-RHex [21]	75	390	570	220	125	120	175	9.5
XRL [22]	100	405	510	205	110	110	175	9.5
Abhis [28]	100	380	560	220	115	115	180	9.2
IRHex [32]	135	410	540	N/A	N/A	N/A	N/A	12.5
CLHeRo	80	650	800	245	120	80	160	9.3
CLHeRo V2.5	80	650	555	245	160	160	200	10.0

**Table 3:** Computer components.

Component	Model
Processor	Intel i7 8700
Memory	Kingston HyperX DDR4@2400 8GB
Hard disk	SSD m.2 256GB
Motherboard	Asus Prime H310i mini-Itx
Power supply	M4-ATX-HV 6-34V
Sink	Scythe Kodati rev-B

**Table 4:** Error results for the simulated forward tests.

# Test	MSE [m^2^]	Maximum Error [m]
1	0.0001	0.0352
2	0.0001	0.0501
3	0.0006	0.0524
4	0.0014	0.0652
5	0.0010	0.0643
6	0.0008	0.0747
7	0.0019	0.3258
8	0.0030	0.1005
9	0.0039	0.1059
10	0.0014	0.0797

**Table 5:** CLHeRo parameters for tests at indoor scenario 1.

Parameter	Value
Legs diameter	160 [mm]
Ground sweep angle	60 [degrees]
Ground rotation speed	1 [rad/s]
Flight rotation speed	5 [rad/s]

**Table 6:** Error results for the real forward tests.

# Test	MSE [m^2^]	Maximum Error [m]
1	0.1802	0.0122
2	0.2130	0.0344
3	0.3025	0.0194
4	0.1416	0.0029
5	0.0824	0.0019
6	0.0376	0.0002
7	0.0753	0.0017
8	0.0702	0.0008
9	0.0283	0.0001
10	0.0452	0.0003
11	0.1115	0.0052
12	0.0985	0.0025
13	0.1017	0.0028
14	0.1005	0.0028
15	0.1311	0.0000
16	0.0334	0.0001

**Table 7:** Results for the indoor scenario 2 test 1: Walking straight. Error units (m).

		Trial				
		1	2	3	4	5
ORB-SLAM2	X	0.0184	0.0013	0.0238	0.0070	0.0007
	Y	0.1931	0.1176	0.2293	0.4302	0.0755
	Z	0.0438	0.0438	0.0746	0.0727	0.0874
T265	X	0.7464	0.8530	0.6511	0.3196	0.2189
	Y	0.1446	0.0196	0.2640	0.3068	0.1052
	Z	0.1561	0.2085	0.0434	0.1170	0.1641
Odometry	X	0.1449	0.0866	0.0611	0.0440	0.0663
	Y	0.1247	0.4174	0.1024	0.2769	0.1866
	Z	0.0430	0.0438	0.0487	0.0456	0.0561

**Table 8:** Results for the indoor scenario 2 test 2: Walking with different ground sweep angle.

		Trial		
		30°	45°	60°
ORB-SLAM2	X	0.0184	0.0013	0.0238
	Y	0.1931	0.1176	0.2293
	Z	0.0438	0.0438	0.0746
T265	X	0.7464	0.8530	0.6511
	Y	0.1446	0.0196	0.2640
	Z	0.1561	0.2085	0.0434
Odometry	X	0.1449	0.0866	0.0611
	Y	0.1247	0.4174	0.1024
	Z	0.0430	0.0438	0.0487
EKF	X	0.1449	0.0866	0.0611
	Y	0.1247	0.4174	0.1024
	Z	0.0430	0.0438	0.0487
Odometry + IMU	X	0.1449	0.0866	0.0611
	Y	0.1247	0.4174	0.1024
	Z	0.0430	0.0438	0.0487
Odometry + ORB SLAM2	X	0.1449	0.0866	0.0611
	Y	0.1247	0.4174	0.1024
	Z	0.0430	0.0438	0.0487
IMU + ORB SLAM2	X	0.1449	0.0866	0.0611
	Y	0.1247	0.4174	0.1024
	Z	0.0430	0.0438	0.0487

**Table 9:** Visual odometry systems, mean error measurements.

Angle [degrees]	Angle [rads]	X Error [m]	Y Error [m]
45	π/4	−0.1	0.15
90	π/2	−0.3	0.2
135	3π/4	−0.45	0.15
180	pi	−0.6	0.0
225	5π/4	−0.45	−0.25
270	3π/2	−0.3	−0.35
315	7π/4	−0.05	−0.25

**Table 10:** Vertical average distance.

Angle [degrees]	Angle [rads]	Vertical Displacement [cm]
45	0.79	1.2
30	0.52	1.6
22.5	0.39	2.1
11.25	0.2	2.7

**Table 11:** Translational error for turn in place test. Angle units—degrees. Error units—m.

		Trial							
		T2		T3		T4		T5	
		X	Y	X	Y	X	Y	X	Y
ORB-SLAM2	0°	0.0	0.0	0.0	0.0	0.0	0.0	0.0	0.0
	90°	−0.273	0.270	−0.242	0.183	−0.268	0.193	−0.275	0.205
	180°	0.643	0.011	−0.517	−0.003	−0.568	0.008	−0.563	0.004
	270°	−0.296	−0.324	−0.246	−0.286	−0.268	−0.323	−0.276	−0.360
	360°	−0.059	−0.002	0.047	0.000	0.020	0.000	0.008	0.000
T265	0°	0.0	0.0	0.0	0.0	0.0	0.0	0.0	0.0
	90°	−0.270	0.191	−0.256	0.174	−0.265	0.213	−0.263	0.181
	180°	−0.511	0.006	−0.434	−0.002	−0.504	0.008	−0.499	0.004
	270°	−0.303	−0.248	−0.248	−0.230	−0.265	−0.284	−0.262	−0.335
	360°	0.009	0.005	0.034	0.007	0.009	0.002	0.001	0.009
Odom+VO	0°	0.0	0.0	0.0	0.0	0.0	0.0	0.0	0.0
	90°	−0.264	0.212	−0.117	0.085	−0.140	0.092	−0.154	0.084
	180°	−0.327	−0.001	−0.244	−0.003	−0.287	−0.002	−0.324	−0.048
	270°	−0.244	−0.070	−0.098	−0.172	−0.146	−0.173	−0.141	−0.254
	360°	−0.068	0.022	0.032	−0.005	0.148	−0.007	0.004	−0.018
EKF	0°	0.0	0.0	0.0	0.0	0.0	0.0	0.0	0.0
	90°	−0.262	0.157	−0.111	0.081	−0.142	0.093	0.153	0.087
	180°	−0.321	−0.001	−0.236	−0.017	−0.287	−0.027	−0.337	−0.074
	270°	−0.251	−0.059	−0.097	−0.138	−0.142	−0.174	−0.135	−0.249
	360°	−0.080	0.015	0.037	−0.005	0.004	−0.017	0.004	−0.020

**Table 12:** Configuration parameters for the indoor circuit test.

		Trial			
	Units	1	2	3	4
Turn	Angle [rads]	0.39	0.39	0.39	0.20
	Speed [rads/s]	0.50	0.50	0.50	0.10
Straight	Angle [rads]	1.05	1.05	1.05	1.05
	Speed [rads/s]	1.00	1.00	1.00	1.00

**Table 13:** Final errors for the indoor scenario 2 test 3: Complete a circuit. Error units—m.

		Trial			
		1	2	3	4
ORB-SLAM2	X	0.0269	0.0469	2.2719	0.0037
	Y	0.2226	0.1527	2.3802	0.1026
	Z	0.0098	0.0782	0.0329	0.0081
T265	X	0.0352	0.0233	0.0018	0.0261
	Y	0.2367	0.0756	0.0251	0.1034
	Z	0.0625	0.0058	0.0067	0.0011
Odometry	X	0.1654	0.7119	1.0992	1.9157
	Y	1.5035	2.6927	3.6621	3.5649
	Z	0.0085	0.0074	0.1154	0.1102
EKF	X	0.1048	0.1424	0.285	0.07
	Y	0.1618	0.1129	1.4407	0.1013
	Z	0.1174	0.2355	0.0858	0.3018
Odometry + ORB SLAM2	X	0.0668	0.145	0.0743	0.0868
	Y	0.0336	0.0881	2.9011	0.2136
	Z	0.0099	0.0782	0.0017	0.0081
IMU + ORB SLAM2	X	0.0248	2.0957	2.8439	2.2643
	Y	0.2216	0.2475	0.0885	0.4837
	Z	0.0066	0.2001	0.0878	0.2025

**Table 14:** Results for the outdoor scenario 2 test 1: Walking straight.

		Final Position [m]		
		X	Y	Z
	Paved road	10.75	0.37	0.00
	Uneven terrain 1	10.10	1.17	–
	Uneven terrain 2	9.8	1.22	–

**Table 15:** Results for the outdoor scenario 1: Walking straight. Error units—m.

		Trial		
		Asphalt	Uneven #1	Uneven #2
ORB-SLAM2	X	0.0404	0.1729	0.3716
	Y	0.0795	0.0773	0.1369
	Z	0.0106	0.1135	0.2352
T265	X	0.0471	1.6320	2.2178
	Y	0.0868	0.3560	0.1082
	Z	0.3424	0.0570	0.0676
Odometry	X	0.0667	1.8340	1.8043
	Y	0.3700	1.1700	1.17
	Z	0.0000	0.0000	0.0000
EKF	X	0.4730	0.7388	0.9753
	Y	0.1990	0.2043	0.4433
	Z	0.0487	0.2161	0.3085
Odometry + IMU	X	0.7727	1.8332	1.8038
	Y	0.7868	2.0644	1.6895
	Z	0.0000	0.0007	0.0001
Odometry + ORB SLAM2	X	0.0163	0.6670	0.8209
	Y	0.0822	0.3524	0.2330
	Z	0.0116	0.1123	0.2348
IMU + ORB SLAM2	X	0.0405	0.2252	0.3718
	Y	0.0795	0.1045	0.1366
	Z	0.0118	0.1006	0.2345
